# Calcium Ions Aggravate Alzheimer’s Disease Through the Aberrant Activation of Neuronal Networks, Leading to Synaptic and Cognitive Deficits

**DOI:** 10.3389/fnmol.2021.757515

**Published:** 2021-12-02

**Authors:** Pei-Pei Guan, Long-Long Cao, Yi Yang, Pu Wang

**Affiliations:** College of Life and Health Sciences, Northeastern University, Shenyang, China

**Keywords:** calcium ions, transporters, mechanisms, Alzheimer’s disease, review

## Abstract

Alzheimer’s disease (AD) is a neurodegenerative disease that is characterized by the production and deposition of β-amyloid protein (Aβ) and hyperphosphorylated tau, leading to the formation of β-amyloid plaques (APs) and neurofibrillary tangles (NFTs). Although calcium ions (Ca^2+^) promote the formation of APs and NFTs, no systematic review of the mechanisms by which Ca^2+^ affects the development and progression of AD has been published. Therefore, the current review aimed to fill the gaps between elevated Ca^2+^ levels and the pathogenesis of AD. Specifically, we mainly focus on the molecular mechanisms by which Ca^2+^ affects the neuronal networks of neuroinflammation, neuronal injury, neurogenesis, neurotoxicity, neuroprotection, and autophagy. Furthermore, the roles of Ca^2+^ transporters located in the cell membrane, endoplasmic reticulum (ER), mitochondria and lysosome in mediating the effects of Ca^2+^ on activating neuronal networks that ultimately contribute to the development and progression of AD are discussed. Finally, the drug candidates derived from herbs used as food or seasoning in Chinese daily life are summarized to provide a theoretical basis for improving the clinical treatment of AD.

## Introduction

Alzheimer’s disease (AD) is a neurodegenerative disease with cognitive deficit as the main characteristic (Elgh et al., 2006). During the course of AD development and progression, calcium ion (Ca^2+^) concentrations are obviously increased in the brains of patients with AD and APP/PS1 Tg mice (Cao et al., 2019). One report has shown that β-amyloid protein (Aβ)_1–40_ has the ability to increase Ca^2+^ influx in rat cortical synaptosomes and cultured cortical neurons (MacManus et al., 2000). Similar to Aβ_1–40_, Aβ_1–42_ induce the Ca^2+^ influx *via* RyRs in primary cultured hippocampal neurons (Marcantoni et al., 2020). Furthermore, the Aβ_25–35_ peptide promotes Ca^2+^ influx by activating L- and T-type Ca^2+^ channels in rat hippocampal slices (Li et al., 2010). The APP intracellular domain (AICD), a APP cleavage fragment, may act as a transcription factor to activate the Ca^2+^ signaling system (Cao and Südhof, 2001; Leissring et al., 2002). Because of the self-aggregating characteristics of Aβ, Aβ oligomers can promote Ca^2+^ influx through N-methyl-D-aspartic acid receptor (NMDAR) channels in a short period of time (Kelly and Ferreira, 2006). More directly, Arispe et al. (2010) found that the aggregates of Aβ_1–40_ and Aβ_1–42_ form a cation channel on the surface of an artificial lipid membrane that allows the passage of Ca^2+^. The pore formation ability of Aβ was confirmed and corroborated by atomic force microscopy (Lin et al., 2001), electron microscopy (Lashuel et al., 2002, 2003), and a theoretical model (Durell et al., 1994; Jang et al., 2008).

Reciprocally, Ca^2+^ is not a passive contributor to the development and progression of AD. In PS-mutant AD brain tissue, a Ca^2+^ metabolic disorder was evident before the formation of APs or NFTs (Etcheberrigaray et al., 1998), which indicated that the metabolic disorder caused by Ca^2+^ located in the cytoplasm might be the cause of AD. Based on this hypothesis, previous studies have shown that Ca^2+^ influx increases the production and aggregation of Aβ and the phosphorylated tau protein, which affects the learning and memory of patients with AD (Etcheberrigaray et al., 1998; Zempel et al., 2010; Tong et al., 2018). Moreover, Ca^2+^ imbalance leads to dysregulated metabolism that affects many neurophysiological functions related to AD, including the regulation of neuroinflammation, response to neuronal injury, neuronal regeneration, neurotoxicity and autophagy (Wahlestedt et al., 1993; Liu and Zukin, 2007; Decuypere et al., 2011a; Sama and Norris, 2013; Song et al., 2019). These actions of Ca^2+^ may finally contribute to neuronal death, which results in cognitive decline during the course of AD development and progression.

Given the multiple functions of Ca^2+^ in AD, its transporters in the cell membrane, endoplasmic reticulum (ER), mitochondria and lysosomes must be involved in regulating the development and progression of AD. As an antagonist of NMDAR, a Ca^2+^ transporter on the surface of the nerve cell membrane, memantine significantly inhibits Ca^2+^ influx and was the first Food and Drug Administration (FDA)-approved drug for the treatment of moderate to severe AD in patients (Bullock, 2006). Regarding the important reservoir of Ca^2+^ in neurons, the ER has been reported to release Ca^2+^ to the cytosol, which contributes to the development and progression of AD (Guan et al., 2021). Although direct evidence showing the relationship between Ca^2+^ transport from mitochondria and lysosomes and the learning ability of patients with AD is unavailable, voltage-dependent anion channel protein 1 (VDAC1) is a hub protein that interacts with phosphorylated tau, Aβ, and γ-secretase, and it contributes to their toxic effects on triggering cell death and potentially leading to the dementia that is a characteristic of AD (Shoshan-Barmatz et al., 2018). All this evidence prompted us to summarize the roles of Ca^2+^ transporters located in different organelles in regulating the development and progression of AD.

Therefore, this review mainly summarizes the molecular mechanisms by which a Ca^2+^ imbalance in individuals with AD affects the regulation of neuroinflammation, neuronal injury, neuronal regeneration, neurotoxicity, neuroprotection, and autophagy, specifically from the perspective of Ca^2+^ transporters in the cell, mitochondria, endoplasmic reticulum and lysosomal membranes. By addressing these mechanisms, we will fill the gaps between increased Ca^2+^ concentrations and the fate of neurons, which results in dementia.

## Crosstalk Between Factors Responsible for Ca^2+^ Dyshomeostasis and Neuroinflammation

### Ca^2+^ Increases the Production of Proinflammatory Cytokines

Neuroinflammation is widely accepted to be mediated by Ca^2+^ dyshomeostasis and induces the cognitive decline associated with AD. This process is studied to understand the inherent mechanisms by which Ca^2+^ exerts an effect. For example, Ca^2+^ increases the production of interleukin (IL)-1β and tumor necrosis factor α (TNF-α) *via* calcineurin (CaN) in glial cells (Sama and Norris, 2013). Consistently, an indirect blockade of Ca^2+^ entry into lipopolysaccharide (LPS)-activated microglia stimulates the production of proinflammatory cytokines, such as TNF-α and IL-6 (Dolga et al., 2012). These observations revealed critical roles for Ca^2+^ in inducing neuroinflammation by concurrently increasing the production of proinflammatory cytokines and decreasing the levels of anti-inflammatory cytokines.

### Transporters on the Cell Membrane Mediate the Effects of Ca^2+^ on the Secretion of Proinflammatory Cytokines

Based on these observations, Ca^2+^ transporters were found to be involved in regulating neuroinflammation. More specifically, NMDAR is critical for mediating the effects of Ca^2+^ on stimulating the production of proinflammatory cytokines, such as IL-1β and TNF-α, in primary mouse hippocampal neurons and lamina II neurons of isolated spinal cord slices (Kawasaki et al., 2008; Huang et al., 2011). By deactivating NMDAR, sevoflurane, an NMDAR antagonist, inhibits the production of IL-1β, TNF-α, IL-6, and IL-8, whereas the addition of the NMDAR agonist D-cycloserine restores the suppression of ageing phenotype acquisition in rats (Yang Z. Y. et al., 2020). NMDAR overexpression in primary cultured microglial cells was induced to synthesize nitric oxide (NO) by activating the NF-κB signaling pathway and to exclude the nonspecific action of these pharmacological interventions (Murugan et al., 2011). In the context of inflammation, NMDAR blockade attenuates the clinical symptoms of glutamate excitotoxicity, suggesting that NMDAR exerts potential neuroprotective effects (Wallström et al., 1996). Similar to this observation, blocking the AMPA/kainate receptor also results in the neuroprotection of encephalomyelitis-sensitized mice (Pitt et al., 2000; Smith et al., 2000). Based on this observation, researchers have readily deduced that α-amino-3-hydroxy-5-methyl-4-isoxazole propionate receptor (AMPAR) might also be involved in regulating neuroinflammation. In SG neurons and lamina II neurons isolated from spinal cord slices, AMPAR was reported to mediate Ca^2+^-stimulated secretion of proinflammatory cytokines, such as IL-1β and TNF-α (Liu et al., 2013). Perampanel, an AMPAR antagonist, concurrently suppressed the expression of proinflammatory cytokines, including IL-1β and TNF-α, and upregulates the expression of anti-inflammatory cytokines, including IL-10 and Transforming Growth Factor Beta 1 (TGF β1), in a rat model of traumatic brain injury (TBI; Chen T. et al., 2017).

**Figure 1:**
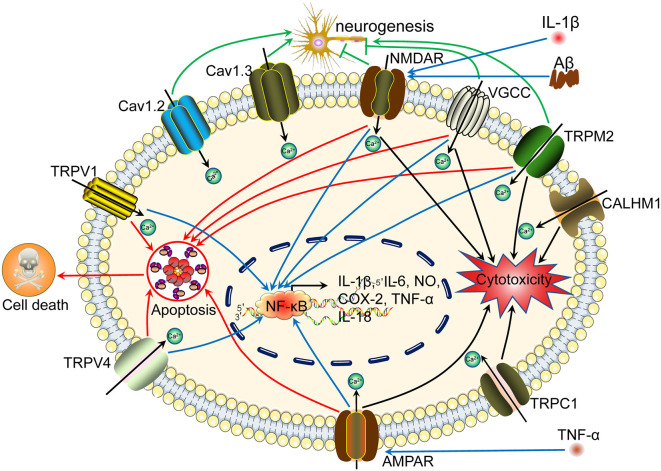
Ca^2+^ participates in regulating neuroinflammation, neuronal injury, neurogenesis, neurotoxicity, neuroprotection, autophagy and apoptosis *via* its transporters located on the cell membrane. Aβ activates Ca^2+^ transporters, including NMDAR, AMPAR, LTCC, Na ^+^/K ^+^ -ATPase, CALHM1, TRPV1, and Cav1.2, which promote Ca^2+^ entry into the cytoplasm and increase the concentration of Ca^2+^ in neuronal cells. More importantly, these Ca^2+^ transporters mediate the effects of Ca^2+^ on neuroinflammation, neuronal injury, neurogenesis, neurotoxicity, neuroprotection, autophagy, and apoptosis through different mechanisms. Aβ activates NMDAR, LTCC, CALHM1, and TRPV1, which result in apoptosis induction, leading to cell death. Regarding neuroinflammation, NMDARs mediate the effects of Aβ on activating NF-κB through a Ca^2+^-dependent mechanism, which results in transcriptional regulation of the secretion of IL-1β, IL-6, NO, and TNF-α. Moreover, NMDARs induce LC3 II production, leading to autophagy.

In addition to glutamate receptors serving as transporters of Ca^2+^, some Ca^2+^ transporters in the cell membrane are reported to be involved in regulating neuroinflammation. For example, the blockade of L-type voltage-gated calcium channels (L-VGCC) by bepridil, nitrendipine or nimodipine attenuates neuroinflammation by deactivating astrocytes and microglial cells in LPS-stimulated or artificial cerebrospinal fluid (aCSF)-injected (i.c.v.) rats and astrocytes from the CA1 region of the hippocampus (Brand-Schieber and Werner, 2004; Daschil et al., 2013; Espinosa-Parrilla et al., 2015; Hopp et al., 2015). These observations were corroborated by the ability of Ca^2+^ to induce TNF-α production in cultured rat hippocampal neurons through an L-VGCC-dependent mechanism (Furukawa and Mattson, 1998). In addition, transient receptor potential channels (TRPs) have been identified in mammals and are grouped into six families associated with the onset of neurodegenerative diseases of the central nervous system (CNS): vanilloid TRP (TRPV), melastatin TRP (TRPM), ankyrin TRP (TRPA), polycystin TRP (TRPP), and canonical or classical TRP (TRPC) channels (Morelli et al., 2013). Among these channels, TRPM2 deletion suppresses cytokine production by deactivating microglial cells in TRPM2-knockout mice (Miyanohara et al., 2018; Kakae et al., 2019). Activation of the TRPV1 channel increases the production of proinflammatory cytokines, such as IL-6, in microglial cells (Sappington and Calkins, 2008). The roles of TRPV4 in inflammation are still being debated. By blocking TRPV4 channels, the release of IL-1β and TNF-α is inhibited because of the reduced Ca^2+^ influx, leading to the attenuation of glial cell-mediated inflammation (Shi et al., 2013). In contrast, the opening of TRPV4 channels by a selective TRPV4 agonist, 4α-phorbol 12, 13-didecanoate (4α-PDD), prevents microglial activation and TNF-α release after LPS treatment, and TRPV4 knockdown eliminates the inhibitory effect of agonists on the release of TNF-α from cultured microglial cells (Konno et al., 2012). According to these findings, TRPV4 activation may be induced by microglial cell swelling after activation with LPS. Channel activation may thus serve as an autoregulator to avoid excess microglial activation. In addition, TRPC1-mediated negative regulation may exert an immunosuppressive effect by blocking the initiation of inflammatory pathways in primary microglial cells (Sun Y. et al., 2014; Figure 1). Although Apolipoprotein E4 (APOE4) is not regarded as a canonical Ca^2+^ transporter, human APOE4 increases the activity of microglial cells by inducing the expression of IL-1β in E4F AD mice (Rodriguez et al., 2014). In contrast to APOE4, other isoforms of APOEs inhibit the synthesis of inflammatory mediators, including COX-2, PGE_2_, and IL-1β, in primary cultured microglia obtained from the adult rat brain cortex (Chen et al., 2005).

### The Endoplasmic Reticulum Is Involved in Regulating the Production of Proinflammatory Cytokines and Represents Intracellular Ca^2+^ Stores

Regarding intracellular stores, genetic ablation of type 2 inositol 1,4,5-triphosphate receptor (InsP3R2) increases the production of cytokines in SOD1^G93A^ mice (Staats et al., 2016). By blocking the activity of Ryanodine Receptor (RyR) with dantrolene, the secretion of inflammatory markers is attenuated because of the deactivation of microglia in LPS-infused rats (Hopp et al., 2015). Treatment with PK11195, a mitochondrial ligand, inhibits store-operated calcium entry (SOCE)-mediated Ca^2+^ influx, resulting in the downregulation of COX-2 expression in human microglial cells (Hong et al., 2006). Thus, the endoplasmic reticulum (ER), as an intracellular Ca^2+^ store, is critical for regulating neuroinflammation *via* InsP3R-, RyR- and SOCE-dependent mechanisms. Interferon α/β (IFNα/β) induce cell apoptosis through Ca^2+^ release-activated Ca^2+^ (CRAC; Yue et al., 2012). As an important component of the mitochondrial permeability transition pore (mPTP), cyclophilin (CypD) knockdown decreases the secretion of proinflammatory cytokines, including Vascular Cell Adhesion Molecule 1 (VCAM-1), IL-6 and TNF-α, in the arteries of mice (Liu et al., 2019; Figure 3).

**Figure 2:**
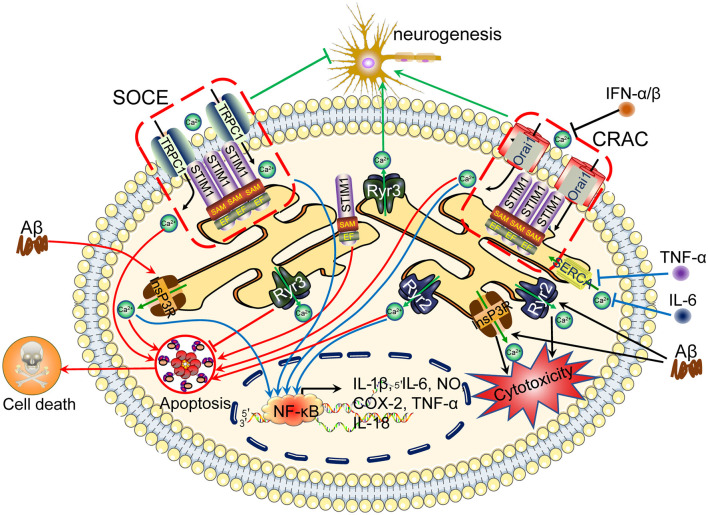
Ca^2+^ channels in the ER are involved in regulating neuroinflammation, apoptosis, tau phosphorylation and Aβ deposition, leading to cognitive impairment. The accumulation of Aβ in neuronal cells induces Ca^2+^ influx from the intracellular Ca^2+^ store, namely, the ER. In addition, Ca^2+^ depletion from the ER triggers sustained extracellular Ca^2+^ influx to the cytosol *via* a SOCE pathway, including TRPC1 and Orai1, by activating the Stim. During these processes, InsP3R and RyR2 play important roles in inducing Ca^2+^ influx from the ER to the cytosol, regulating apoptosis, neurogenesis, tau phosphorylation and Aβ deposition and subsequently leading to cognitive impairment. ER, endoplasmic reticulum; SOCE, store-operated calcium entry.

**Figure 3:**
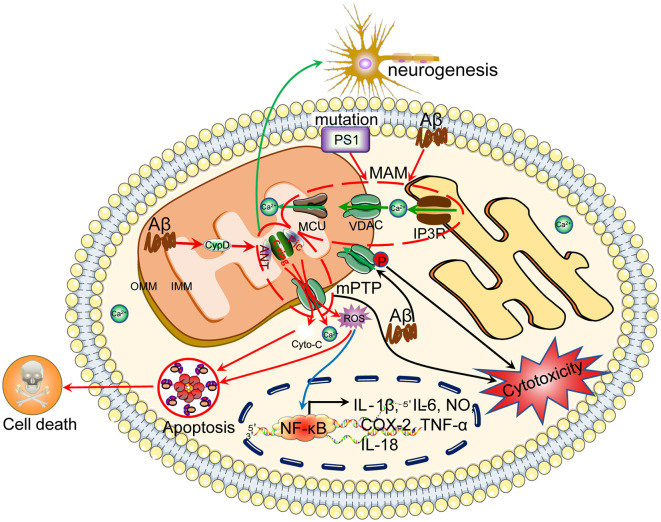
Ca^2+^ efflux from mitochondria regulates the apoptosis of neuronal cells, which results in cognitive dysfunction. Ca^2+^ is transported to the mitochondria *via* MCU. Under physiological or pathological conditions, Ca^2+^ is continuously shuffled between the ER and mitochondria *via* VDAC. Moreover, Ca^2+^ in mitochondria induces the formation of the mPTP, which transports Ca^2+^ and small molecules, such as ROS and cytochrome C, from the mitochondria to the cytosol, leading to neuronal apoptosis. The loss of neurons will cause cognitive dysfunction.

With opposite effects, proinflammatory cytokines have the ability to modulate the Ca^2+^ balance *via* their transporters. For example, TNF-α, IL-1β, and IFNγ increase the influx of Ca^2+^ into microglial cells, which indicates crosstalk between Ca^2+^ and neuroinflammatory factors in cultured hippocampal neurons (Goghari et al., 2000; McLarnon et al., 2001; Franciosi et al., 2002). IL-1β increases the expression of AMPAR on the cell surface, which potentially contributes to the entry of Ca^2+^ into hippocampal neurons (Viviani et al., 2003; Simões et al., 2012). In contrast to AMPAR, IL-1β inhibits L-VGCC activity by suppressing the protein expression of Ca^2+^ channels in primary cultured neurons (Zhou et al., 2006; Zhou, 2010). In addition, IL-1β is responsible for increasing the expression of TRPM2, leading to the influx of Ca^2+^ to microglial cells (Fonfria et al., 2006). Similar to IL-1β, IL-6 potentiates Ca^2+^ entry through NMDARs in hippocampal neurons (Orellana et al., 2005). Although IL-6 is not expressed in neuronal cells, it downregulates the expression of SERCA2, which blocks Ca^2+^ entry into the ER, thus maintaining high levels of cytosolic Ca^2+^ in cardiac myocytes (Villegas et al., 2000). Similar to other cytokines, TNF-α increases Ca^2+^ currents through NMDARs in cultured rat hippocampal neurons (Furukawa and Mattson, 1998). In addition, TNF-α induces the rapid insertion of AMPAR into the membranes of hippocampal pyramidal neurons (Ogoshi et al., 2005). In addition, the colocalization of GluA1, GluA2 and GluA4 and synaptophysin on the neural crest also indicates the transportation of AMPAR to synapses (Wigerblad et al., 2017). In contrast, TNF-α decreases Ca^2+^ influx by inhibiting the activity of L-VGCCs in cultured rat hippocampal neurons and hippocampal CA1 neurons (Furukawa and Mattson, 1998; Sama et al., 2012). Regarding the regulation of intracellular stores, impaired TNF-α signaling disrupts the effects of InsP3R on mediating Ca^2+^ release from the ER to the cytosol in 3xTg mice (Park et al., 2010). Moreover, calcineurin (CaN) is activated by the proinflammatory cytokine TNF-α in astrocytes (Fernandez et al., 2007; Sama et al., 2008; Furman et al., 2012). TNF-α activates a more complicated mechanism to regulate Ca^2+^ currents. In addition to TNF-α itself, the TNF-α receptor mobilizes Ca^2+^ through an RyR-dependent mechanism in cultured neonatal rat dorsal root ganglion (DRG) neurons (Pollock et al., 2002). In addition to proinflammatory cytokines, most investigations have focused on the roles of anti-inflammatory cytokines on Ca^2+^ transporters. Based on this information, researchers also found that anti-inflammatory cytokines, such as IL-10, reduced the intracellular Ca^2+^ levels in microglial cells by decreasing Ca^2+^ release from the ER through the deactivation of the InsP3R-dependent mechanism in cultured hippocampal neurons (Turovskaya et al., 2012). Therefore, the existence of crosstalk between Ca^2+^ and neuroinflammation will result in the aggravation of AD (Figure 2).

### Proinflammatory Cytokines Reciprocally Regulate the Activities of Transporters Expressed on Lysosomes to Regulate the Basal Ca^2+^ Levels in Glial Cells

In SH-SY5Y cells, IFNγ also induces Ca^2+^ influx by activating TRPM2, leading to the apoptosis of cultured neurons (Sama et al., 2012). Furthermore, IFNγ reduces the activity of ATPase Sarcoplasmic/Endoplasmic Reticulum Ca^2+^ Transporting 2b (SERCA2b) in IL-1β-stimulated OSCC cells (Cardozo et al., 2005; Gkouveris et al., 2018). In addition to these cytokines, inflammatory factors, such as H_2_O_2_, increase TRPM2 activity, which might lead to increased basal Ca^2+^ levels in cultured rat microglial cells (Kraft et al., 2004). Poly ADP-ribose polymerase-1 (PARP-1) induces Ca^2+^ influx by activating TRPM2 in PARP-2 knockout mice (Kraft et al., 2004). All this evidence revealed crosstalk between Ca^2+^ and neuroinflammatory factors, which aggravates AD *via* the actions of different transporters (Table 1).

**Table 1:** Crosstalk between Ca^2+^ dysregulation and neuroinflammation.

Cat.	Stimulator/Mediator	Mechanism	Experimental model	References
Ca^2+^	CaN	Ca^2+^→IL-1β and TNF-α	Glial cells	Sama and Norris (2013)
	CyPPA	LPS→Ca^2+^→TNF-α and IL-6	Primary mouse microglial cells	Dolga et al. (2012)
CM	NMDAR	NMDAR→Ca^2+^→IL-1β and TNF-α	Primary mouse hippocampal neurons and lamina II neurons of isolated spinal cord slices	Kawasaki et al. (2008) and Huang et al. (2011)
		Sevoflurane ⊣ NMDAR→IL-1β/-6/-8 and TNF-α D-cycloserine→NMDAR→IL-1β/-6/-8 and TNF-α	Ageing rats	Yang Z. Y. et al. (2020)
		NMDAR→NF-κB→NO	Primary microglial cells	Murugan et al. (2011)
	AMPAR	AMPAR→Ca^2+^→IL-1β and TNF-α	SG neurons and lamina II neurons of isolated spinal cord slices	Kawasaki et al. (2008), Park et al. (2008) and Liu et al. (2013)
		Perampanel ⊣ AMPAR→IL-1β and TNF-α ∪ ⊣ IL-10 and TGF-β1.	TBI model in rats	Chen T. et al. (2017)
	L-VGCC	Bepridil, nitrendipine and nimodipine ⊣ L-VGCC→astrocytes and microglia cells→neuroinflammation	Encephalomyelitis (EAE)-induced multiple sclerosis (MS) animal model; LPS or aCSF-injected (i.c.v) rats; astrocytes in the CA1 region of the hippocampus	Brand-Schieber and Werner (2004), Daschil et al. (2013), Espinosa-Parrilla et al. (2015), and Hopp et al. (2015)
		L-VGCC→Ca^2+^→TNF-α	Rat hippocampal neurons	Furukawa and Mattson (1998)
	TRPM2	TRPM2^−/–^ ⊣ microglial cells→cytokines	TRPM2^−/–^ mice	Miyanohara et al. (2018) and Kakae et al. (2019)
	TRPV1	TRPV1→IL-6	Microglial cells	Sappington and Calkins (2008)
	TRPV4	Blocking TRPV4 channels ⊣ Ca^2+^ influx→IL-1β and TNF-α→inflammation	Glial cells	Shi et al. (2013)
		4α-phorbol 12, 13-didecanoate (4α-PDD)→TRPV4 ⊣ microglial activation→TNF-α	Rat microglial cells	Konno et al. (2012)
	TRPC1	TRPC1→microglia-mediated inflammation	Primary microglial cells	Sun Y. et al. (2014)
	APOE4	hAPOE4→IL-1β→microglia cells	E4F AD mice	Rodriguez et al. (2014)
	APOEs	APOE1–3 ⊣ COX-2, PGE_2_ and IL-1β	Primary microglial cells from the rat brain cortex	Chen et al. (2005)
ER	InsP3R2	InsP3R2^−/–^→cytokines	SOD1^G93A^ mice	Staats et al. (2016)
	RyR	Dantrolene ⊣ RyR→deactivation of microglia→inflammatory markers	LPS-infused rats	Hopp et al. (2015)
	SOCE	PK11195, a mitochondrial ligand ⊣ SOCE→Ca^2+^ influx→COX-2	Human microglial cells	Hong et al. (2006)
MD		CypD→mPTP→IL-6 ∪ TNFα	CypD KO mouse	Liu et al. (2019)
LM		PS1/2^−/–^→Ca^2+^ efflux from lysosomes	PS1/2^−/–^ MEFs	Coen et al. (2012) and McBrayer and Nixon (2013)
IL-1β/TNF-α/IFNγ	Ca^2+^	TNF-α, IL-1β, and IFNγ→Ca^2+^ influx	Microglial cells	Goghari et al. (2000), McLarnon et al. (2001), and Franciosi et al. (2002)
IL-10	InsP3R	IL-10 ⊣ InsP3R→Ca^2+^ efflux from the ER	Hippocampal neurons	Turovskaya et al. (2012)
IL-1β	NMPAR	IL-1β→NMPAR→Ca^2+^ influx	Hippocampal neurons	Viviani et al. (2003) and Simões et al. (2012)
	L-VGCC	IL-1β ⊣ Ca^2+^ channels→L-VGCC	Primary neurons	Zhou et al. (2006) and Zhou (2010)
	TRPM2	IL-1β→TRPM2→Ca^2+^ influx	Human C13 microglia cells	Fonfria et al. (2006)
IL-6	NMDAR	IL-6→NMDAR→Ca^2+^ influx	Hippocampal neurons	Orellana et al. (2005)
	SERCA	IL-6 ⊣ SERCA	Cardiac myocytes	Villegas et al. (2000)
TNF-α	NMDAR	TNF-α→NMDAR→Ca^2+^ currents	Rat hippocampal neurons	Furukawa and Mattson (1998)
	TRPM2			
	CP-AMPAR	TNF-α→CP-AMPAR	Hippocampal neurons	Ogoshi et al. (2005)
		TNF-α→GluA1	Male Holtzman rats	Wigerblad et al. (2017)
	TRPM2	IFNγ→TRPM2→Ca^2+^ influx	SH-SY5Y cells	Güzel et al. (2021)
	L-VGCC	TNF-α ⊣ L-VGCC→Ca^2+^ influx	Rat hippocampal neurons and hippocampal CA1 neurons	Furukawa and Mattson (1998) and Sama et al. (2012)
	InsP3R	TNF-α^−/–^ ⊣ InsP3R→Ca^2+^ efflux from the ER	3xTg mice	Park et al. (2010)
	Calcineurin	TNF-α→CaN	Astrocytes	Fernandez et al. (2007), Sama et al. (2008), and Furman et al. (2012)
TNFα	RyR	TNFα→RyR→Ca^2+^ mobilization	Neonatal rat DRG neurons	Pollock et al. (2002)
IFNγ/LPS	TRPM2	IFNγ and LPS→TRPM2→Ca^2+^ influx	Microglial cells in TRPM2^−/–^ mice	Miyake et al. (2014)
IL-1β/IFNγ	SERCA2b	IL-1β and IFNγ ⊣ SERCA2b	Pancreatic cells	Cardozo et al. (2005)
		IFNγ ⊣ SERCA2b	Human OSCC cell line	Gkouveris et al. (2018)
H_2_O_2_	TRPM2	H_2_O_2_→TRPM2→Ca^2+^ influx	Rat microglial cells	Kraft et al. (2004)
		PARP1→TRPM2→Ca2 + influx	PARP1 KO mice	Raghunatha et al. (2020)

## Ca^2+^ Signaling Impairs Neuronal Function

### The Effects of Ca^2+^ on Impairing Neuronal Functions

Given the crosstalk between Ca^2+^ and neuroinflammatory factors, we continued to elucidate the roles of Ca^2+^ in impairing neuronal functions and its effects on the relationship between neuroinflammation and neuronal apoptosis and death (Table 2). For example, accumulating evidence has revealed that appropriate activation of microglial cells may exert beneficial effects by attenuating neuronal apoptosis, increasing neurogenesis, and promoting functional recovery after cerebral ischaemia (Neumann et al., 2008). In contrast, overactivation of microglial cells may result in the apoptosis or death of neurons (Brown and Neher, 2014). Based on these findings, excessive release of Ca^2+^ initially protects neuronal cells from death by inducing the expression of Bcl-2 through the activated transcription factor NF-κB (Pahl and Baeuerle, 1996; Mattson and Furukawa, 1997), whereas sustained increases in cytosolic Ca^2+^ concentrations induced by neuronal depolarization result in Aβ_1–42_ production and subsequent neuronal death (Pierrot et al., 2004). Moreover, a series of studies reviewed in our previous work described the effects of Ca^2+^ on cell apoptosis *via* multiple signaling pathways, and this information is not repeated in the present review (Wang and Wang, 2017).

**Table 2:** The effect of Ca^2+^ on impairing neuronal functions.

Cat.	Stimulator or Mediator	Mechanism	Experimental model	References
Ca^2+^		Ca^2+^→NF-κB→Bcl-2 ⊣ neuronal death	Primary rat hippocampal neurons	Pahl and Baeuerle (1996) and Mattson and Furukawa (1997)
		Ca^2+^→Aβ_1–42_→neuronal death	Rat cortical neurons	Pierrot et al. (2004)
		XeC ⊣ Aβ_1–42_→IP3→Ca^2+^→apoptosis	Primary hippocampal neurons	Wang et al. (2019)
CM	NMDAR	IL-1β→NMDAR→Ca^2+^ influx→neuronal apoptosis	Rat hippocampus	Dong et al. (2017)
		IL-1β→NMDAR ∪ tyrosine phosphorylation→neuronal death	Co-culture of primary hippocampal neurons and glial cells	Viviani et al. (2006)
		IL-6 ⊣ NMDAR→Ca^2+^→JAK/CaN →neuronal death	Cerebellar granule neurons (CGNs)	Ma et al. (2015)
	AMPAR	TNF-α→trafficking GluR2-lacking AMPARs to the plasma membrane→cell death	Spinal cord neurons	Ferguson et al. (2008) and Beattie et al. (2010)
	L-VGCC	Gas6 ⊣ L-VGCC→Aβ-induced apoptosis	Cortical neurons	Yagami et al. (2002)
		Nimodipine ⊣ L-VGCC→Ca^2+^ influx→Aβ-induced neuronal apoptosis	Primary cortical and hippocampal neurons	Ueda et al. (1997) and Yagami et al. (2002)
		PFHxS→NMDAR ∪ L-VGCC→AMPK ∪ ERK→apoptosis	PC12 cells	Lee et al. (2016)
	TRPV1	TRPV1^+/+^→mitochondria→cytochrome c→cell death	Human microglia cell line (HMO6)	Kim et al. (2006) and Zhang and Liao (2015)
	TRPV4	TRPV4^+/+^→neuronal apoptosis	Rats with neuronal injury	Shi et al. (2013)
		TRPV4^−/–^ ⊣ IL-1β and TNF-α→neuronal cell death	Glial cells	Shi et al. (2013)
		TRPV4^−/–^ ⊣ infrasound-induced neuronal death	Rat microglial cells	Konno et al. (2012)
	TRPM2	TRPM2→Ca^2+^→neuronal death	Rat insulinoma RIN-5F cells and rat cortical neurons	Kaneko et al. (2006)
		TRPM2 siRNA ⊣ Aβ-induced neuronal death	Primary rat neurons	Fonfria et al. (2005)
	APOE4	APOE4→Ca^2+^ influx→neuronal death	SH-SY5Y cells	Veinbergs et al. (2002)
		APOE4→NMDAR ∪ CaMKII→apoptosis	APOE^−/–^ mice and primary cultures of cerebral cortical neurons from APOE^−/–^ mice	Xu and Peng (2017)
		APOE4 overexpression→Ca^2+^ influx→neuronal apoptosis	APOE4-expressing neurons	Jiang et al. (2015)
ER		TBI→APOE4→apoptosis	Tg mice overexpressing human APOE4/APOE3	Giarratana et al. (2020)
	UPR	ER stress→UPR→cell apoptosis	Prion protein-infected mice	Moreno et al. (2013)
	Misfolded proteins	Misfolded proteins accumulate→ER stress→Ca^2+^ influx→apoptosis	Patients with AD, PD and ALS	Nishitoh et al. (2009)
	InsP3R	InsP3R3→Ca^2+^ efflux from the ER→cell death	Postnatal cerebellar granule cells	Blackshaw et al. (2000)
		Isoflurane→InsP3R→caspase-3→apoptosis	DT40 cells	Joseph et al. (2014)
		P2X7R, isoflurane and sulforaphane→InsP3R-mediated Ca^2+^ efflux from the ER→apoptosis or cell death	NG108–15 and PC12 neurons and nude mice	Wei et al. (2008), Chao et al. (2012), and Hudecova et al. (2016)
		Aβ_25–35_→InsP3R→Ca^2+^ efflux from the ER→apoptosis of astrocytes	Murine astrocytes	Oseki et al. (2014)
	RyR	S-gluthathionylation→RyR2^PMT^→cortical neuronal death	Rats with cerebral ischaemia	Bull et al. (2008)
		RyR3 suppression→neuronal death	TgCRND8 neurons	Supnet et al. (2010)
	Stim1	Stim1^−^ ⊣ H_2_O_2_-induced apoptosis	Endothelial progenitor cells	Wang et al. (2016)
		Stim1 siRNA ⊣ Ca^2+^ influx ⊣ neuronal viability ∪ →apoptotic cell death	*In vitro* traumatic neuronal injury	Hou et al. (2015)
	Stim1/Orai	Resveratrol (RSV) ⊣ Stim1 and Orai1 ⊣ autophagic cell death	PC3 and DU145 cells	Selvaraj et al. (2016)
		Stim1^−^ and Orai^−^ ⊣ SOCE→LPS-induced apoptosis	Pulmonary microvascular endothelial cells	Wang et al. (2016)
	Orai	Orai1^mut^ ⊣ SOCE and thapsigargin-induced apoptosis	Human prostate cancer (PCa) cells	Flourakis et al. (2010)
MT		Curcumin ⊣ mitochondrial damage from oxidative stress→neuronal apoptosis	Rat cortical neurons	Zhu et al. (2004)
		Sal→mitophagy ⊣ apoptosis	Primary cultures of spinal neurons	Gu et al. (2020)
	Aβ	Aβ_1–42_→Drp1 ∪ ⊣ Mfn1/2 and OPA-1→neuronal apoptosis	Primary mouse cortical neurons	Han et al. (2017)
		Aβ_25–35_→mitochondria→cytochrome c→apoptosis	NT2 cells	Morais Cardoso et al. (2002)
	mPTP	InsP3R→Ca^2+^→mPTP→cytochrome c→cell apoptosis	HepG2 cells	Szalai et al. (1999)
		CBD→mPTP→ROS→cytochrome c→apoptosis	Human monocytes	Wu et al. (2018)
		Mortalin^+^ ⊣ mPTP→Aβ-induced neuronal apoptosis	SH-SY5Y cells	Qu et al. (2012)
		CyPD^−/–^ ⊣ mPTP→cell death	mAPP mice	Du et al. (2008)
	VDAC	VDAC1^+^→Ca^2+^→cell death and apoptosis	A549 cells	Weisthal et al. (2014)
		Antibody ⊣ VDAC1→Aβ induced neuronal apoptosis	Hippocampal neurons	Thinnes (2011)
		VDAC→cell apoptosis	Lymphoblastoid cells carrying the mitochondrial DNA mutation	Yuqi et al. (2009)
		VDAC→cytochrome c ∪ Bax→permeating membranes	VDAC1-deficient mitochondria from a mutant yeast	Shimizu et al. (1999)
		Caspase-8→cleaves Bid→VDAC closure→protein release from mitochondria→apoptosis	Planar phospholipid membranes	Rostovtseva et al. (2004)
		Bcl-xL→VDAC open ⊣ apotosis.	FL5.12 cells	Vander Heiden et al. (2001)
		FABP5→VDAC1 ∪ BAX→apoptosis	Human KG-1C oligodendroglial cells	Cheng et al. (2020)
		BAPTA-AM ⊣ Ca^2+^→VDAC1 oligomerization→ mitochondria-mediated apoptosis	HeLa or T-REx-293 cells	Keinan et al. (2013)
		DIDS, SITS, H2DIDS, DNDS, and DPC ⊣ VDAC1 oligomerization→apoptosis	VDAC1 ^+^ HeLa cells	Ben-Hail and Shoshan-Barmatz (2016)
		DIDS ⊣ VDAC1→Ca^2+^→apoptosis	THP-1 macrophages	Chen et al. (2014)

### Proinflammatory Cytokines Induce Neuronal Apoptosis or Death *via* Ca^2+^ Transporters Located on the Cell Membranes

However, transporters have not been considered critical for mediating the effects of Ca^2+^ on the apoptosis or death of neurons. Therefore, we further addressed the roles of different types of Ca^2+^ transporters in regulating the apoptosis or death of neuronal cells, especially during the course of AD development and progression. Due to its close association with neuroinflammation, neuronal apoptosis in the rat hippocampus is induced by IL-1β through an NMDAR-mediated Ca^2+^ influx mechanism (Dong et al., 2017). By coculturing glial cells with primary hippocampal neurons, IL-1β secreted from glial cells triggers neuronal death *via* tyrosine phosphorylation and NMDAR trafficking mechanisms (Viviani et al., 2006; Dong et al., 2017). In contrast to the action of IL-1β, IL-6 reduces Ca^2+^ overload by deactivating NMDARs, which resulted in the death of cultured cerebellar granule neurons (CGNs) *via* the JAK/CaN pathways (Ma et al., 2015). As another type of glutamate receptor involved in Ca^2+^ transport, AMPAR, which is trafficked to the plasma membrane, mediates the effects of TNF-α on exacerbating the effects of spinal cord injury on cell death (Ferguson et al., 2008; Beattie et al., 2010). By inhibiting the activities of L-VGCC, Gas6 or nimodipine suppresses Aβ-induced neuronal apoptosis by attenuating Ca^2+^ influx into primary cultured cortical and hippocampal neurons (Ueda et al., 1997; Yagami et al., 2002). In addition, NMDARs and L-VGCCs mediate the effects of perfluorohexanesulfonate (PFHxS) on activating the AMPK and ERK pathways, leading to the apoptosis of P12 cells (Lee et al., 2016). Among the Ca^2+^ transporters located in the cell membrane, TRPV1 overexpression disrupts mitochondrial function and induces cytochrome c release, which results in the death of a human microglial cell line (HMO6; Kim et al., 2006; Zhang and Liao, 2015). Similarly, ectopically expressed TRPV4 in glial cells induces neuronal damage *via* an apoptotic mechanism (Shi et al., 2013). Consistent with these findings, pharmacological or genetic interventions targeting TRPV4 suppress neuronal cell death by decreasing the expression of proinflammatory cytokines, such as IL-1β and TNF-α (Konno et al., 2012; Shi et al., 2013). As another type of TRP family protein, TRPM2 is activated to induce Ca^2+^ influx, resulting in the death of RIN-5F rat insulinoma cells and rat cortical neurons (Kaneko et al., 2006). TRPM2 knockdown reduces the toxicity of Aβ and subsequent death of primary rat neuron cultures (Fonfria et al., 2005; Li and Jiang, 2018; Figure 1).

### Ca^2+^ Transporters Located on the ER Membrane Are Responsible for Regulating Neuronal Apoptosis

Although APOE4 is not a canonical Ca^2+^ transporter, APOE4 overexpression induces Ca^2+^ influx, resulting in neuronal apoptosis (Veinbergs et al., 2002; Jiang et al., 2015). Through a more complicated mechanism, APOE4 induces neuronal apoptosis in APOE4 knockout mice by activating NMDAR-mediated Calcium/Calmodulin dependent protein kinase II (CaMKII) pathways (Qiao et al., 2017). Moreover, TBI induces apoptosis in the cortex and hippocampus of Tg mice overexpressing human APOE4 by activating APOE4 (Giarratana et al., 2020). In addition, ER stress also mediates the effects of the unfolded protein response (UPR) and misfolded proteins on inducing apoptosis through mechanisms related to Ca^2+^ influx (Nishitoh et al., 2009; Moreno et al., 2013). Specifically, Ca^2+^ transporters located on the ER membrane, including InsP3R and RyR, are reported to be involved in regulating neuronal apoptosis. For example, type 3 InsP3R regulates cell death by modulating Ca^2+^ release from the ER to the cytosol in postnatal cerebellar granule cells (Blackshaw et al., 2000; Wang and Zheng, 2019). Isoflurane treatment induces Ca^2+^ influx, leading to caspase-3 activation by cleavage in DT40 cells (Joseph et al., 2014). Upon the stimulation of P2X7R by isoflurane and sulforaphane, InsP3R mediates the effects of Ca^2+^ on inducing apoptosis or cell death of NG108-15 and PC12 neuronal cells and cells in nude mice (Wei et al., 2008; Chao et al., 2012; Hudecova et al., 2016). Specifically, Aβ_25–35_ induces the apoptosis of murine astrocytes *via* InsP3R- and Ca^2+^-activating pathways (Oseki et al., 2014). In addition to InsP3R, the posttranslational modification of RyR2 by S-glutathionylation increases channel activity, resulting in the death of rat cortical neurons (Bull et al., 2008). In contrast, the suppression of RyR3 expression in TgCRND8 neurons increases the neuronal death rate, which suggests a protective role for RyR in the late stages of AD pathogenesis (Supnet et al., 2010).

Based on these observations, ethanol dose-dependently increases the intracellular Ca^2+^ concentration, which damages HepG2 hepatocytes by upregulating the expression of the Orai1 and Stromal interaction molecule 1 (Stim1) mRNAs and proteins (Liu et al., 2012). Although the pathophysiological effects of decreased Store-operated calcium entry (SOCE) levels in AD remain unclear, several lines of evidence have shown that SOCE deficits lead to neuronal cell death and decreased synaptic plasticity (Soboloff and Berger, 2002; Calvo-Rodriguez et al., 2020). As expected, Stim1 silencing alleviates the apoptosis of H_2_O_2_-treated endothelial progenitor cells (Wang et al., 2016). Moreover, the downregulation of Stim1 by an siRNA concurrently increases neuronal viability and inhibits apoptotic cell death by decreasing the intracellular Ca^2+^ levels (Selvaraj et al., 2016). In PC3 and DU145 cells, both Stim1 and Orai1 separately mediate the effects of resveratrol (RSV), a natural polyphenol, on activating autophagic cell death (Selvaraj et al., 2016). In addition, resveratrol can mediate the release of Ca^2+^ from intracellular stores (Santoro et al., 2020). As a method to exclude nonspecific effects of pharmacological interventions, silencing the expression of Stim1 and Orai1 reduces the apoptosis rate of LPS-treated pulmonary microvascular endothelial cells by blocking SOCE in pulmonary microvascular endothelial cells (Wang et al., 2016). Researchers excluded the effects of Stim1 on cell apoptosis by transfecting Orai1 mutants and observed decreases in both SOCE and the rate of thapsigargin-induced apoptosis in human prostate cancer (PCa) cells (Flourakis et al., 2010; Figure 2).

### Mitochondrial Dysfunction Is Also Involved in Mediating the Effects of Ca^2+^ on Neuronal Apoptosis

However, ER stress is not the only mechanism by which the effects of Ca^2+^ on neuronal apoptosis are mediated: mitochondrial dysfunction is also reported to be involved in this process (Yoon et al., 2011). Consistently, Stim1 or Orai1 knockdown attenuates the intracellular Ca^2+^ overload, restores the mitochondrial membrane potential, decreases the release of cytochrome c and inhibits ethanol-induced apoptosis (Cui et al., 2015). Without affecting ER stress, curcumin protects mitochondria from oxidative damage by attenuating the apoptosis of cortical neurons (Zhu et al., 2004). In primary cultured spinal neurons, salidroside (Sal) treatment decreases apoptosis by activating PINK-Parkin pathways, leading to mitophagy of mitochondria (Gu et al., 2020). Similar to its effects on AD, Aβ_1–42_ induces neuronal apoptosis by concurrently upregulating mitochondrial fission protein dynamin-related protein 1 (Drp1) and downregulating mitofusin 1/2 (Mfn1/2) and dynamin-like GTPase (OPA-1) in primary cultures of mouse cerebral cortical neurons (Han et al., 2017). In addition, Aβ_25–35_ induces cytochrome c-mediated apoptosis of NT2 cells through a functional mitochondria-dependent mechanism (Morais Cardoso et al., 2002). In this mechanism, Ca^2+^ transport by InsP3R to mitochondria induced by opening the mPTP induces the release of cytochrome c, which results in the apoptosis of cells (Szalai et al., 1999). In fact, mPTP opening induces matrix swelling, the subsequent rupture of the outer membrane, and nonspecific release of proteins in the intermembrane space into the cytosol upon cannabidiol (CBD) induction of human monocyte apoptosis (Wu et al., 2018). By inhibiting the opening of the mPTP in mitochondria, mortalin overexpression blocks Aβ-induced SH-SY5Y cell apoptosis (Qu et al., 2012). In AD mice, CyPD knockout decreases the cell death rate by attenuating the opening of the mPTP in mitochondria (Du et al., 2008; Pahrudin Arrozi et al., 2020).

VDAC1 expression induces cell death and apoptosis by activating the Ca^2+^ signaling cascade in A549 cells (Weisthal et al., 2014). VDAC is involved in the apoptosis of lymphoblastoid cells carrying a mitochondrial DNA mutation (Yuqi et al., 2009). Through a direct interaction with Bax, VDAC induces the transport of cytochrome c through membranes (Shimizu et al., 1999). Moreover, the cleavage of the pro-apoptotic protein Bid by caspase-8 induces the closure of VDAC, which leads to protein release from mitochondria and apoptosis (Rostovtseva et al., 2004). In contrast, Bcl-xL promotes the opening of the VDAC, which results in a reduced apoptosis rate of cultured FL5.12 cells (Vander Heiden et al., 2001; Bessou et al., 2020). Fatty acid binding protein 5 (FABP5), which is expressed in oligodendrocytes, induces mitochondrial macropore formation through VDAC-1 and Bax, thus accelerating mitochondria-induced glial cell death. These two proteins mediate mitochondrial outer membrane permeability, resulting in the release of mitochondrial DNA and cytochrome c into the cytoplasm and activation of apoptotic caspases (Cheng et al., 2020). More interestingly, BAPTA-AM, a Ca^2+^-chelating reagent, inhibits mitochondria-mediated apoptosis by decreasing the oligomerization of VDAC1 in HeLa and T-REx-293 cells (Keinan et al., 2013). Consistent with this observation, anion transport inhibitors, including 4’-diisothiocyano-2,2’-stilbenedisulfonic acid (DIDS), SITS, H_2_DIDS, DNDS, and DPC, inhibit apoptosis-associated VDAC1 oligomerization (Ben-Hail and Shoshan-Barmatz, 2016). In addition, blockade of plasmalemmal VDAC1 with a specific antibody suppresses Aβ-induced neuronal apoptosis (Thinnes, 2011; Lim et al., 2021a). In THP-1 macrophages, DIDS disodium salt, an inhibitor of VDAC1, attenuates the apoptosis of THP-1 macrophages by decreasing intracellular Ca^2+^ levels (Chen et al., 2014). Similarly, Ca^2+^ transporters generally mediate the regulatory effects of Ca^2+^ on neuronal apoptosis, especially in the context of AD (Figure 3).

## Ca^2+^ Inhibits The Regulation of Neuronal Stem Cells

### Ca^2+^ Modulates Neuronal Differentiation, Migration and Self-renewal During the Course of Neurogenesis

During the course of AD development and progression, neurogenesis is markedly inhibited in the brains of patients with AD and mouse models (Rash et al., 2016). Given the potential roles of Ca^2+^ in AD, we summarize the effects of Ca^2+^ on neurogenesis during the course of AD development and progression. Indeed, higher frequencies of Ca^2+^ oscillations increase the differentiation of hippocampus-derived neural stem cells (NSCs) into neurons in adult rats (Wang Q. et al., 2015). Moreover, Epac2 mediates PACAP-induced differentiation of neural progenitor cells (NPCs) into astrocytes along with an increase in intracellular Ca^2+^ levels, which also activated the signaling pathway for astrocytogenesis in Epac2-knockout (KO) mice (Seo and Lee, 2016). NSC differentiation is closely related to the expression of VGCC, especially Caveolin 1 (Cav1) through regulating Ca^2+^ influx (D’Ascenzo et al., 2006). Moreover, exposure in extremely low-frequency electromagnetic fields (ELFEF) promotes the differentiation of NSCs by upregulating the expression and function of Cav1 (Piacentini et al., 2008c). Furthermore, bidirectional radial Ca^2+^ activity elongates the fiber of radial glial cells (RGCs) and simultaneously induces neurogenesis during early cortical column development (Rash et al., 2016). By upregulating the Notch signaling pathway after brain injury, Ca^2+^ waves generated in neighboring astrocytes propagate to NPCs, inducing neurogenic behavior, including the self-renewal and migration of progenitor cells (Kraft et al., 2017). Based on these observations, Ca^2+^ induces neuronal differentiation, migration and self-renewal during the course of neurogenesis.

### Ca^2+^ Transporters Located on the Cell Membranes Are Required for Neurogenesis

Given the key roles of Ca^2+^ in neurogenesis, its transporters are also required for neurogenesis. In the developing cerebellum, granule cell precursors differentiate upon activation of a homodimeric G protein-coupled receptor that is sensitive to Ca^2+^ levels called calcium-sensing receptor (CaSR). CaSR activation *in vivo* induces the homing of granule cell precursors during differentiation, mainly through CaSR interactions with integrin complexes (Tharmalingam et al., 2016). Among these CaSRs, the lower activity of NMDARs in NR1^+/–^ mice contributes to increased cell proliferation and neurogenesis compared to the activity in the brains of adult NR1^+/+^ mice (Bursztajn et al., 2007). In contrast, intraperitoneal injection of the NMDAR agonist NMDA (2 mg/kg/day) promotes cell proliferation in the subventricular zone (SVZ) of rats (Fan et al., 2012). Unfortunately, the researchers did not extend their investigations to Ca^2+^, although NMDAR affects neurogenesis. Compared to NMDARs, the roles of AMPARs in neurogenesis are relatively simple. In rats administered chronic corticosterone (CORT), S47445, a novel AMPAR-positive allosteric modulator (AMPA-PAM), exerted significant neurogenic effects on the proliferation, survival and maturation of new hippocampal neurons (Mendez-David et al., 2017). Moreover, AMPAR mediates kainate-induced radial glia-like stem cell proliferation (Shtaya et al., 2018). Human NPCs contain Ca^2+^-permeable AMPARs; however, AMPARs were engineered to become Ca^2+^-impermeable receptors during the course of differentiation from NPCs to neurons or astrocytes through RNA editing of the AMPA receptor subunit GluR2 at the Q/R site (Whitney et al., 2008). Then, the NMDAR subunits NR1 and NR2B and the AMPAR subunit GluR2 in Ca^2+^-impermeable AMPARs were upregulated at the mRNA level in differentiated neuroepithelial precursors, indicating their likely contribution to neurotransmission after first establishing neuronal networks (Muth-Köhne et al., 2010; Wang et al., 2018).

In addition to NMDARs and AMPARs, different types of VGCCs and TRPs in cell membranes are also involved in regulating neurogenesis. For example, the differentiation of dental pulp stem cells (DPSCs) into neural cells is markedly inhibited by regulating the levels of the distal C-terminus (DCT) upon treatment with nimodipine and knock down of Cav1.2 expression (Ju et al., 2015). In the dentate gyrus (DG) region, deletion of Cav1.2 decreases the numbers of doublecortin-positive adult-born neurons, suggesting important roles for Cav1.2 in adult neurogenesis (Temme et al., 2016). Consistent with these findings, Cav1.3 knockout impairs hippocampal neurogenesis and inhibits neuronal differentiation (Marschallinger et al., 2015). More importantly, Ca^2+^ mediates the effects of L-VGCC on the neurogenesis of interneurons in nifedipine-treated NPCs (Brustein et al., 2013). Similar to L-VGCCs, blockade of other types of VGCCs, such as N- and T-VGCCs, decreases the migration and neurite extension of developing neurons (Komuro and Rakic, 1992; Louhivuori et al., 2013). On the other hand, TRPs are also reported to be involved in regulating neurogenesis. For instance, TRPM2 deficiency results in impaired embryonic neurogenesis because it regulates neural progenitor self-renewal through an SP5-dependent mechanism (Li and Jiao, 2020). In addition, the antisense oligonucleotide-mediated knockdown of TRPC1 expression reduces the effects of bFGF on the proliferation of embryonic rat NSCs (Fiorio Pla et al., 2005; Toth et al., 2016). Blocking SOCE activity with YM-58483 (BPT2) decreases the proliferation of SVZ and neural stem cells (Domenichini et al., 2018). By stereotactically injecting a recombinant adeno-associated virus expressing TRPC1 into the DG of the bilateral hippocampus, we observed that neurogenesis, LTP induction, and cognitive enhancement related to environmental enrichment (EE) were effectively rescued in TRPC1 knockout mice (Du et al., 2017). Consistent with this observation, TRPC3 knockout reduces the effect of Ca^2+^ on mGluR5-mediated radial glial processes, reducing the neuronal migration rate (Louhivuori et al., 2015; Toth et al., 2016). In addition to these classical Ca^2+^ transporters in the cell membrane, both APOE1–3 knockout and APOE4 overexpression suppress neurogenic responses *in vivo* (Hong et al., 2013; Rijpma et al., 2013; Geffin et al., 2017). Based on this evidence, transporters are involved in mediating the effects of Ca^2+^ on the neurogenesis of NPCs and NSCs (Figure 1).

### Intracellular Ca^2+^ Stores Mediate the Effects of Ca^2+^ on Neurogenesis

The ER and mitochondria are major intracellular Ca^2+^ stores and thus mediate the regulatory effects of Ca^2+^ on neurogenesis. In PC12 cells, ER stress and BDNF-TrkB signaling pathways are involved in the induction of neurogenesis by 3β, 23, 28-trihydroxy-12-oleanene 3β-caffeate from *Desmodium sambuense* (Cheng et al., 2019). In addition, ER stress mediates the effects of tunicamycin and HRD1 deletion on the aberrant induction of neuronal differentiation and inhibition of dendrite outgrowth in retinoic acid-treated P19 mouse embryonic carcinoma cells (Kawada et al., 2014). More interestingly, transcripts encoding the three main isoforms of the two families of intracellular calcium release channels, namely, InsP3R and RyR, were detected during early neurogenesis in the mouse cerebral cortex (Faure et al., 2001). In particular, an antagonist of the InsP3 pathway, wortmannin, prevents neurogenesis in neural crest cells (Evrard et al., 2004). In addition, Ca^2+^ waves propagate through radial glial cells in the proliferative cortical ventricular zone (VZ) and require connexin hemichannels, P2Y1 ATP receptors, and intracellular InsP3-mediated Ca^2+^ release, suggesting critical roles for radial glial signaling mechanisms in cortical neuronal production (Weissman et al., 2004; Lim et al., 2021b). In this process, the G protein-coupled receptor GPR157, an orphan G protein-coupled receptor, is involved in regulating the neuronal differentiation of radial glial progenitors through Gq-InsP3-mediated Ca^2+^ cascades (Takeo et al., 2016). In mesenchymal stem cells, caffeine, an RyR agonist, induces an intracellular Ca^2+^ response that increases throughout neuronal differentiation (Resende et al., 2010). Specifically, RyR2 knockout decreases the neurogenesis of embryonic stem cells (Yu et al., 2008). Associated with the aforementioned mechanisms, the proliferation of embryonic and adult NPCs cultured as neurospheres and progenitors in the subventricular zone (SVZ) of adult mice *in vivo* was attenuated by depleting the expression of Stim1 and Orai1, suggesting pivotal roles for SOCE channel-mediated Ca^2+^ entry in mammalian neurogenesis (Somasundaram et al., 2014). In addition to Orai1, single knock down of Stim1, a Ca^2+^ sensor that mediates SOCE, impairs early and late embryonic stem cell differentiation into neural progenitors, neurons or astrocytes, increasing the cell death rate and suppressing the proliferation of neural progenitors (Hao et al., 2014; Deb et al., 2020). Similarly, pharmacological blockade of SOCE decreases the proliferation and self-renewal of NSCs, driving asymmetric division to the detriment of symmetric proliferative division, reducing the population of stem cells in the adult brain, and impairing the ability of SVZ cells to form neurospheres in culture (Domenichini et al., 2018). CRAC channels serve as a major route of Ca^2+^ entry in NSCs/NPCs and regulate key effector functions, including gene expression and proliferation, indicating that CRAC channels are important regulators of mammalian neurogenesis (Somasundaram et al., 2014). Similar to the ER, mitochondria are intracellular Ca^2+^ stores involved in regulating the neurogenesis of NPCs. For example, the inhibition of mPTPs and a selective reduction in mitochondrial superoxide spikes significantly ameliorates the negative effects of Aβ_1–42_ on NPC proliferation and survival (Hou et al., 2014). Moreover, cyclosporin A inhibits neuronal differentiation by suppressing mPTP opening (Hou et al., 2013; Namba et al., 2020). All these observations confirm the involvement of Ca^2+^ and its transporters in regulating neurogenesis (Table 3).

**Table 3:** Ca^2+^ regulates the neurogenesis of neuronal stem cells.

Cat.	Stimulator or Mediator	Mechanism	Experimental model	References
Ca^2+^		Ca^2+^ oscillations→differentiation	Adult rat NSCs	Wang Q. et al. (2015)
		PACAP→Epac2→Ca^2+^→differentiation	NPCs from Epac2^−/–^ mice	Seo and Lee (2016)
		Ca^2+^→elongate the fibers of radial glial cells (RGCs)→neurogenesis	Mouse embryonic forebrain/radial glial cells	Rash et al. (2016)
		Brain injury→Notch→Ca^2+^→neurogenic behavior, including the self-renewal and migration of neurons	NPCs obtained after permanently occluding the middle cerebral artery of mice	Kraft et al. (2017)
CM	NMDAR	NR1^+/–^ ⊣ NMDAR ⊣ cell proliferation and neurogenesis	NR1^+/–^ vs. NR1^+/+^ mice	Bursztajn et al. (2007)
		NMDA→NMDAR→cell proliferation	Rat subventricular zone (SVZ)	Fan et al. (2012)
	AMPAR	S47445→AMPAR→neurogenic effects on the proliferation, survival and maturation of hippocampal newborn neurons	Chronic CORT-treated rats	Mendez-David et al. (2017)
		Kainate→AMPAR→proliferation	Radial glia (RG)-like stem cells	Shtaya et al. (2018)
	L-VGCC/Cav1.2	Nimodipine ∪ Cav1.2^−^ ⊣ differentiation	Rat DPSCs	Ju et al. (2015)
		Cav1.2^−/–^ ⊣ neurogenesis	Cav1.2^−/–^ mice	Temme et al. (2016)
	L-VGCC/Cav1.3	Cav1.2^−/–^ ⊣ hippocampal neurogenesis and neuronal differentiation	Cav1.3^−/–^ mice	Marschallinger et al. (2015)
	L-VGCC	Nifedipine ⊣ L-VGCC→Ca^2+^→neurogenesis	NPCs	Brustein et al. (2013)
	N-VGCC	Antagonist ⊣ N-VGCC→migration of granule cells	Granule cells	Komuro and Rakic (1992)
	T-VGCC	T-VGCC^−^ ⊣ migration and neurite extensions	Neurosphere cultures of neural progenitor cells	Louhivuori et al. (2013)
	TRPM2	TRPM2^−^ ⊣ embryonic neurogenesis	NSCs	Li and Jiao (2020)
	TRPC1	TRPC1^−^ ⊣ bFGF→proliferation	Rat embryonic NSCs	Fiorio Pla et al. (2005) and Toth et al. (2016)
		BTP2 ⊣ TRPC1→SOCE→proliferation	C57BL/6 mice	Domenichini et al. (2018)
		TRPC1→neurogenesis ∪ ERK/CREB	TRPC1^−/–^ mice	Du et al. (2017)
	TRPC3	TRPC3^−/–^ ⊣ Ca^2+^→mGluR5→neuronal migration	NPCs	Louhivuori et al. (2015) and Toth et al. (2016)
	APOE	APOE_1–3_^−^ ∪ APOE_4_^+^ ⊣ neurogenic responses	C57BL/6 mice	Hong et al. (2013)
		APOE_4_^+^ ∪ APOE_1–3_^−^ ⊣ neurogenesis	Aged APOE_4_-overexpressing and APOE_1–3_ knockout mice	Rijpma et al. (2013)
ER		3β, 23, 28-Trihydroxy-12-oleanene 3β-caffeate from *Desmodium sambuense*→ER stress and BDNF-TrkB signaling pathways→neurogenesis	PC12 cells	Cheng et al. (2019)
		Tunicamycin ∪ HRD1-→ER stress→neuronal differentiation ∪ ⊣ dendrite outgrowth	Mouse embryonic carcinoma P19 cells exposed to retinoic acid	Kawada et al. (2014)
	InsP3	Wortmannin ⊣ InsP3→neurogenesis	Neural crest cells	Evrard et al. (2004)
		P2Y1 ATP receptors ∪ InsP3→Ca^2+^→cortical neuronal production	Embryonic cortical ventricular zone (VZ)	Weissman et al. (2004)
		GPR157→Gq-IP3→Ca^2+^→neuronal differentiation of radial glial progenitors	Mouse neocortices at E13 and P0	Takeo et al. (2016)
	RyR	Caffeine→RyR→Ca^2+^→neuronal differentiation	Mesenchymal stem cells	Resende et al. (2010)
		RyR2^−/–^ ⊣ neurogenesis	Embryonic stem cells	Yu et al. (2008)
	Stim1/Orai1	Stim1^−^ ∪ Orai1^−^ ⊣ SOCE→Ca^2+^→proliferation	NPC neurospheres or NPCs in the SVZ of adult mice	Somasundaram et al. (2014)
	Stim1	STIM1^−^ ⊣ SOCE→embryonic stem cell differentiation into neural progenitors, neurons or astrocytes ∪ ⊣ cell death and suppressing the proliferation of neural progenitors	Embryonic stem cells and neural progenitors	Hao et al. (2014)
		SOCE^−^ ⊣ proliferation and self-renewal of NSCs	Cultured NSCs and NSCs in the SVZ	Domenichini et al. (2018)
MT	mPTP	mPTP^−^ ∪ mitochondrial superoxide flash^−^ ⊣ Aβ_1–42_ ⊣ proliferation and survival of NPC	NPCs	Hou et al. (2014)
		Cyclosporine A ⊣ mPTP→neuronal differentiation	NPCs	Hou et al. (2013)

## The Effects of Ca^2+^ on Neurotoxicity

### Ca^2+^ Induces Excitotoxicity *via* Its Transporters Located on Cell Membranes

Neurotoxicity might be the inherent cause of the Ca^2+^-mediated impairment of neuronal functions. In primary cultured cerebral cortical neurons, increased levels of Ca^2+^ induce excitotoxicity, whereas reduced Ca^2+^ release exerts neuroprotective effects (Frandsen and Schousboe, 1991). As the natural ligand of NMDAR, NMDA induces neurotoxicity by activating NMDAR in cerebellar granule cells (Xia et al., 1995). In addition to its natural ligand, the exposure of neurons to ethanol and glutamate also induces neurotoxicity by activating NMDARs (Thomas and Morrisett, 2000; Miao et al., 2012). Similar to its effect on the AD pathway, Aβ_25–35_ induces neurotoxicity by deactivating the pCRMP2 and NMDAR2B signaling pathways in SH-SY5Y cells (Ji et al., 2019). However, the researchers did not extend their observations to the involvement of Ca^2+^ in neurotoxicity. In cultured cerebellar granule neurons, domoic acid induces neurotoxicity through NMDAR-mediated Ca^2+^ influx (Berman et al., 2002). By blocking NMDAR-mediated Ca^2+^ influx, dantrolene and ionomycin prevent neurotoxicity in cultured rat cortical and retinal ganglion cell neurons (Lei et al., 1992). Drug-induced inhibition of Glutamate ionotropic receptor NMDA type subunit 2A (GluN2A) NMDAR or deletion of the GluN2A subunit gene attenuates the effects of homocysteine on increasing intracellular Ca^2+^ concentrations, leading to neurotoxicity (Deep et al., 2019). In hippocampal neurons, Aβ-induced Ca^2+^ influx mediated by NMDARs leads to calpain-dependent neurotoxicity (Kelly and Ferreira, 2006; Deep et al., 2019). Based on these observations, NMDARs have the ability to mediate Aβ-induced neurotoxicity *via* Ca^2+^-dependent mechanisms. In addition, AMPAR was also reported to be involved in regulating neurotoxicity as another glutamate receptor type functioning as a Ca^2+^ transporter. For example, cannabinoid receptor activation attenuates the effects of TNF-α on the surface localization of AMPAR, which resulted in excitotoxicity in cultured hippocampal neurons (Zhao et al., 2010; Ganguly et al., 2019). AMPAR trafficking to the cell membrane of CNS neurons regulates excitotoxicity induced by TNF-α (Ferguson et al., 2008). TNF-α induces a rapid reduction in AMPAR-mediated Ca^2+^ entry by increasing the expression of the GluR2 subunit on the cell surface, which results in excitotoxicity during the progression of neurodegeneration (Rainey-Smith et al., 2010). Moreover, AMPAR mediated AMPA- and kainite-induced neurotoxicity *via* Ca^2+^ influx mechanisms in cultured rat hippocampal neurons (Ambrósio et al., 2000). In addition, ethanol induces neurotoxicity in hippocampal slices by activating AMPAR (Gerace et al., 2021). Of note, either Aβ or trimethyltin has the ability to induce neuronal death *via* activating L-VGCC, leading to the Ca^2+^ overload (Piacentini et al., 2008a, b). Therefore, NMDARs and AMPARs are critical for inducing neurotoxicity by triggering Ca^2+^ influx.

In the cell membrane, L-VGCC is also involved in mediating AMPA/Zn^2+^-induced neurotoxicity in primary cultured rat cortical neurons (Ambrósio et al., 2000; Lee et al., 2016). In these cells, L-VGCCs were further reported to be critical for iron-induced neurotoxicity (Xu Y. Y. et al., 2020). In cerebral cortical cells, CXCL12 induces neurotoxicity *via* NMDAR and L-VGCC-dependent p38 MAPK activation (Sanchez et al., 2016). By blocking the L/N-type Ca^2+^ channel, cilnidipine protects the retina from neurotoxicity in ischaemia-reperfusion-treated rats (Sakamoto et al., 2009).

Another family of Ca^2+^ transporters, TRPs, was also reported to be involved in regulating neurotoxicity. In primary cultures of mouse DRG neurons, the inhibition of TRPV1 with specific blockers, such as capsaicin or resiniferatoxin, reduces the prooxidant capacity of microglial neurotoxicity (Ma et al., 2009). In addition, TRPV1 mediates vanilloid- and low pH-induced neurotoxicity in cultured rat cortical neurons (Shirakawa et al., 2007; Ertilav et al., 2021). In contrast, the inhibition of TRPV1 by the antagonist capsazepine attenuates its neuroprotective effects, indicating that TRPV1 activation contributes to the survival of rat nigral neurons (Park et al., 2012). To the best of our knowledge, no report has reconciled these conflicting results. With respect to TRPC1, neurotoxicity in SH-SY5Y cells is markedly induced by treatment with 1-methyl-4-phenylpyridinium ion (MPP^+^) through TRPC1-deactivating Ca^2+^-dependent mechanisms (Bollimuntha et al., 2005). TRPC1 overexpression inhibits neurotoxicity by inhibiting the release of cytochrome c and the expression of the Bax and Apaf-1 proteins in SH-SY5Y cells (Morelli et al., 2013). In contrast to TRPC1, TRPC6 deletion attenuates the effects of NMDAR-mediated Ca^2+^ entry, resulting in a disruption of the effect of Ca^2+^ on neurotoxicity in primary cultured neurons (Chen J. et al., 2017). Blocking TRPV4-mediated Ca^2+^ influx reduces the neurotoxicity of paclitaxel to small and medium dorsal root ganglion neurons (Boehmerle et al., 2018). Regarding TRPM2, cisplatin-induced neurotoxicity in primary DRG cells is attenuated by treatment with its antagonist, 2-aminoethoxydiphenyl borate (Chen J. et al., 2017). TRPM2 knockout blocks Aβ oligomer-induced neurotoxicity, which results in impaired memory in APP/PS1 mice (Ostapchenko et al., 2015). In hippocampal neurons, Aβ_1–42_ induces neurotoxicity by activating TRPM2 (Li and Jiang, 2018).

In addition to these canonical Ca^2+^ transporters, decreasing the expression of CALHM1 exerts neuroprotective effects on oxygen and glucose deprivation in hippocampal slices (Garrosa et al., 2020). On the other hand, APOE has been reported to be involved in regulating neurotoxicity. For example, APOE4 promotes the neurotoxicity induced by Aβ aggregation in AD (Ma et al., 1996). Extracellular APOE4 is cytotoxic to human neuroblastoma SK-N-SH cells, and Aβ_1–42_ enhances the cytotoxicity of APOE4. The carboxyl terminal mutation of L279Q, K282A or Q284A decreases the ability of APOE4 to form SDS-stable oligomers and decreases its cytotoxicity. Structural and thermodynamic analyses showed that all three APOE4 mutants contain significantly increased α-helical and β-sheet structures, which resulted in reduced exposure of the hydrophobic surface to the solvent and reduced conformational stability during chemical denaturation (Dafnis et al., 2018). In N2a-APP_695_ cells, APOE4 exacerbates the effects of ethanol on inducing neurotoxicity by increasing oxidative stress and apoptosis (Ji et al., 2019). In contrast, APOE1–3 has been shown to protect primary cultures of rat cortical neurons from the neurotoxic effects of the nonfibrillar C-terminal domain of Aβ (Drouet et al., 2001; Brookhouser et al., 2021). APOE isoforms play different roles in neurotoxicity by modulating Aβ deposition in the mouse brain (Drouet et al., 2001). Ca^2+^ mediates the effects of truncated APOE on neurotoxicity in cultured embryonic rat hippocampal neurons (Tolar et al., 1999). Through these mechanisms, APOE-related neurotoxicity might be a therapeutic target for AD (Marques and Crutcher, 2003; Figure 1).

### The ER Mediates the Effects of Ca^2+^ on Inducing Neurotoxicity as an Intracellular Store

Since Ca^2+^ regulates neurotoxicity *via* transporters located in the cell membrane, the roles of Ca^2+^ derived from intracellular stores in neurotoxicity are further addressed in Table 4. For example, Aβ induces neurotoxicity in cortical neurons *via* an ER-mediated apoptotic pathway (Ferreiro et al., 2006; Goswami et al., 2020). In the spinal cord, Ca^2+^ mediates the effects of ER stress on neurotoxicity (Li et al., 2014). By alleviating ER stress, nicotine suppresses the activity of MPP ^+^ /MPTP associated with neurotoxicity in PC12 cells (Cai et al., 2017). Similar to its role in AD, Aβ induces neurotoxicity in cortical neurons by promoting ER stress (Song et al., 2008).

**Table 4:** The effects of Ca^2+^ on neurotoxicity (including neuroprotection).

Cat.	Stimulator or mediator	Mechanism	Experimental model	References
Ca^2+^		Ca^2+^→excitotoxicity ⊣ neuroprotective effects	Primary cerebral cortical neurons	Frandsen and Schousboe (1991)
CM	NMDAR	NMDA→NMDAR→neurotoxicity	Cerebellar granule cells	Xia et al. (1995)
		Ethanol→NMDAR→neurotoxicity	Hippocampal slices	Thomas and Morrisett (2000)
		glutamate→NMDAR→neurotoxicity	Primary rat retinal neurons	Miao et al. (2012)
		Aβ_25–35_ ⊣ pCRMP2 and NMDAR2B ⊣ neurotoxicity	SH-SY5Y cells	Ji et al. (2019)
		Domoic acid→NMDAR→Ca^2+^ influx→neurotoxicity	Cerebellar granule neurons	Berman et al. (2002)
		Dantrolene and ionomycin ⊣ NMDAR→Ca^2+^ influx→neurotoxicity	Rat cortical and retinal ganglion neurons	Lei et al. (1992)
		Homocysteine→GluN2A-NMDAR Ca^2+^ influx→neurotoxicity	Primary cultured cortical neurons	Deep et al. (2019)
		Aβ→NMDAR→Ca^2+^ influx→calpain→neurotoxicity	Hippocampal neurons	Kelly and Ferreira (2006)
	AMPAR	Cannabinoid receptor ⊣ TNF-α→CM-AMPAR→excitotoxicity	Hippocampal neurons	Zhao et al. (2010)
		TNF-α→AMPAR trafficking→excitotoxicity	Spinal neurons	Ferguson et al. (2008)
		TNF-α→GluR2 ⊣ AMPAR→Ca^2+^→ excitotoxicity→neurodegeneration	Primary mouse motor and cortical neurons	Rainey-Smith et al. (2010)
		AMPA ∪ kainate→AMPAR→Ca^2+^→neurotoxicity	Rat hippocampal neurons	Ambrósio et al. (2000)
		Ethanol→AMPAR→neurotoxicity	Hippocampal slices	Gerace et al. (2021)
	L-VGCC	AMPA/Zn^2+^→L-VGCC→neurotoxicity	Primary rat cortical neurons	Ambrósio et al. (2000)
		Iron→L-VGCC→neurotoxicity	Primary rat ventral mesencephalic neurons	Xu Y. Y. et al. (2020)
		CXCL12→NMDAR ∪ L-VGCC→p38→neurotoxicity	Cerebrocortical cells	Sanchez et al. (2016)
		Cilnidipine ⊣ L/N-type Ca^2+^ channel →neurotoxicity	Retina from ischaemia-reperfusion-treated rats	Sakamoto et al. (2009)
	TRP	Capsaicin or resiniferatoxin ⊣ TRPV1→microglial neurotoxicity	Primary mouse DRG neurons	Ma et al. (2009)
		Vanilloids and low pH→TRPV1→neurotoxicity	Rat cortical neurons	Shirakawa et al. (2007)
		Capsazepine ⊣ TRPV1→neuronal survival	Rat nigral neurons	Park et al. (2012)
	TRPC1	MPP^+^ ⊣ TRPC1→Ca^2+^ influx ⊣ neurotoxicity	SH-SY5Y cells	Bollimuntha et al. (2005)
		TRPC1^+^ ⊣ neurotoxicity→cytochrome c, Bax and Apaf-1	SH-SY5Y cells	Morelli et al. (2013)
	TRPV4	Paclitaxel→TRPV4→Ca^2+^→neurotoxicity	DRG neurons	Boehmerle et al. (2018)
	TRPC6	TRPC6^−^ ⊣ NMDAR→Ca^2+^ influx→ neurotoxicity	Primary neurons	Chen J. et al. (2017)
	TRPM2	2-Aminoethoxydiphenyl borate ⊣ TRPM2→cisplatin→neurotoxicity	Primary DRG neurons	Chen J. et al. (2017)
		TRPM2^−/–^ ⊣ Aβ oligomers→neurotoxicity ⊣ memory	TRPM2^−/–^ APP/PS1 mice	Ostapchenko et al. (2015)
		Aβ_1–42_→TRPM2→neurotoxicity	Hippocampal neurons	Li and Jiang (2018)
	CALHM1	CALHM1−/− ⊣ oxygen and glucose deprivation ⊣ neuroprotective effects	Hippocampal slices from WT Calhm1^+/+^, Calhm1^+/–^, and Calhm1^−/–^ mice	Garrosa et al. (2020)
	APOE	APOE4→Aβ aggregates→neurotoxicity→AD	Human cortical neurons	Ma et al. (1996)
		APOE4→Aβ42→neurotoxicity	SK-N-SH cells	Dafnis et al. (2018)
		APOE4 ∪ ethanol→oxidative stress and apoptosis→neurotoxicity	N2a-APP_695_ cells	Ji et al. (2019)
		APOE_2–3_ ⊣ non-fibrillar C-terminal domain of Aβ→neurotoxicity	Primary rat cortical neurons	Drouet et al. (2001)
		APOE isoforms→Aβ→neurotoxicity	Mouse brain	Hudry et al. (2013)
		Truncated APOE→Ca^2+^ influx→neurotoxicity	Embryonic rat hippocampal neurons	Tolar et al. (1999)
		APOE→neurotoxicity→AD	Embryonic rat hippocampal neurons	Marques and Crutcher (2003)
ER		Aβ→ER→apoptotic pathway→neurotoxicity	Cortical neurons	Ferreiro et al. (2006)
		Ozone (O_3_) →ER→Ca^2+^ influx→neurotoxicity	Spinal cord neurons	Li et al. (2014)
		Nicotine ⊣ MPP ^+^ /MPTP→ER stress→neurotoxicity	PC12 cells	Cai et al. (2017)
		Sevoflurane→ER stress→neurotoxicity	Neuronal cells	Komita et al. (2013)
		Aβ→ER stress→neurotoxicity	Cortical neurons	Song et al. (2008)
	IP3	Cyanide→IP3→neurotoxicity	PC12 cells	Yang et al. (1996)
		M3 muscarinic receptors→IP3→Ca^2+^→cytotoxicity	Rat cerebellar granule cells	Limke et al. (2004)
		Microcystin-LR→PLC ∪ IP3→Ca^2+^→neurotoxicty	Hippocampal neurons	Cai et al. (2015)
	InsP3R	Isoflurane ∪ APP^mut^→InsP3R→Ca^2+^ influx→neurotoxicity	SH-SY5Y cells	Liu et al. (2016)
	InsP3R/RyR	Aβ→InsP3R ∪ RyR→Ca^2+^ efflux from the ER→neurtoxicity	Primary cortical cells	Ferreiro et al. (2004)
		InsP3R ∪ RyR→cytotoxicity	PS1^L286V^ mutant PC12 cells	Yang et al. (2019)
	RyR	RyR→neurotoxicity	Human microglial and THP-1 cells	Klegeris et al. (2007)
		Xbpls ⊣ Aβ→RyR→neurotoxicity	Mammalian neurons	Fernandez-Funez et al. (2010)
MT	VDAC	Aβ→VDAC1→neurotoxicity→AD	PC12 and SH-SY5Y cells	Smilansky et al. (2015)
		Hesperidin ⊣ Aβ ⊣ p-VDAC1 ⊣ neurotoxicity	PC12 cells	Wang et al. (2013)
		Aβ ⊣ p-VDAC1 ⊣ neurotoxicity	Murine septal SN56, SH-SY5Y and hippocampal HT22 cells	Fernandez-Echevarria et al. (2014) and Shoshan-Barmatz et al. (2018)
		VDAC ∪ mERα→Aβ-induced neurotoxicity	SN56 and hippocampal HT22 cells	Marin et al. (2007)
		Antibody ⊣ VDAC2→intracellular Ca^2+^→neurotoxicity	SH-SY5Y cells	Marin et al. (2007)
	mPTP	Cyclosporin A ⊣ mPTP→neurotoxicity	SH-SY5Y and PC12 cells	Ye et al. (2016)
		4-Hydroxy-2(E)-nonenal ∪ NMDA→mPTP→Ca^2+^ influx→neurotoxicity	Primary rat cortical neurons	Choi et al. (2013)
		NMDA→mPTP→neurotoxicity	Mouse cortical neurons	Kinjo et al. (2018)

As Ca^2+^ mediates the effects of ER stress on neurotoxicity, Ca^2+^ transporters in ER membranes must be associated with neurotoxicity. For example, The generation of InsP3 by activated M3 muscarinic receptors contributes to increased Ca^2+^ influx and subsequent cytotoxicity in rat cerebellar granule cells (Limke et al., 2004). Furthermore, cyanide induces the formation of InsP3, which triggers intracellular neurotoxic signaling events in PC12 cells (Yang et al., 1996). In hippocampal neurons, Ca^2+^ was also found to be the critical cause of microcystin-LR-induced neurotoxicity through PLC- and InsP3-dependent pathways (Cai et al., 2015). Regarding the receptors of InsP3, InsP3R triggers Ca^2+^ influx to mediate isoflurane-induced neurotoxicity, which is facilitated by an APP mutant in SH-SY5Y cells (Liu et al., 2016). In primary cultures of cortical cells, Aβ induces neurotoxic effects by inducing Ca^2+^ release from the ER *via* InsP3R- and RyR-dependent mechanisms (Ferreiro et al., 2004). After inhibiting the activity of InsP3R and RyR, the cytotoxicity and increased Ca^2+^ levels are attenuated. More interestingly, the combined inhibition of both receptors paradoxically increases the amount of cytosolic Ca^2+^ entering PC12 cells from the extracellular space, increasing cytotoxicity (Yang et al., 2019). In addition to InsP3R, RyR alone might be critical for modulating neurotoxicity in human microglia and THP-1 cells (Klegeris et al., 2007; Holland and Pessah, 2021). In cultured mammalian neurons, Xbpls ameliorates Aβ-induced neurotoxicity through an RyR-dependent mechanism (Fernandez-Funez et al., 2010). Thus, the ER is an important intracellular Ca^2+^ store for regulating neurotoxicity in neurons (Figure 2).

### Mitochondria Are Critical for Regulating Neurotoxicity Through a Ca^2+^-Dependent Mechanism

In addition to the ER, mitochondria are reported to be critical for regulating neurotoxicity through a Ca^2+^-dependent mechanism. In particular, VDAC1, a transporter located in mitochondria, mediates Aβ-induced neurotoxicity in PC12 and SH-SY5Y cells and thus represents a potential target for AD treatment (Smilansky et al., 2015). In addition, the dephosphorylation of VDAC1 by hesperidin blocks Aβ-induced neurotoxicity in PC12 cells through a mitochondria-dependent mechanism (Wang et al., 2013). Aβ directly induces neurotoxicity *via* the dephosphorylation of VDAC1 in murine septal SN56, SH-SY5Y and hippocampal HT22 cells (Fernandez-Echevarria et al., 2014; Shoshan-Barmatz et al., 2018). In these cells, the interaction between VDAC and mERα at the plasma membrane may lead to the modulation of Aβ-induced neurotoxicity (Marin et al., 2007). In addition to VDAC1, an anti-VDAC2 antibody reduces neurotoxicity by decreasing intracellular Ca^2+^ levels in SH-SY5Y cells (Marin et al., 2007; Nagakannan et al., 2019). By inhibiting the opening of the mPTP, cyclosporin A protects SH-SY5Y and PC12 cells from neurotoxicity (Ye et al., 2016). In primary cultures of rat cortical neurons, 4-hydroxy-2(E)-nonenal facilitates NMDA-induced neurotoxicity by opening the mPTP, which results in Ca^2+^ influx (Choi et al., 2013). This observation is further supported by a report showing that NMDA induced neurotoxicity *via* the mPTP in cultured murine cortical neurons (Kinjo et al., 2018). Based on this evidence, intracellular Ca^2+^ stores are involved in mediating the effects of Ca^2+^ on neurotoxicity, which potentially contributes to neuronal apoptosis or death (Table 4, Figure 3).

## Ca^2+^ Disrupts The Autophagic Clearance of Aggregated Proteins

### Ca^2+^ Transporters on the Cell Membranes Are Presumably Involved in Regulating Autophagy and Are Responsible for Clearing Aβ or Phosphorylated Tau

As a protein clearing function, autophagy deficiency might be the cause of the aggregation and deposition of Aβ or hyperphosphorylation of tau in APs and NFTs (Pickford et al., 2008; Heckmann et al., 2019). Ca^2+^ signaling plays a crucial role in autophagy in various experimental models (Shaikh et al., 2016; Zhang et al., 2016). Logically, Ca^2+^ transporters are proposed to be involved in regulating autophagy. According to preliminary evidence, NMDARs on the cell membrane contribute to autophagy and the membrane potential in leukaemic megakaryoblasts (Nursalim, 2016). Specifically, exposure to low-dosage NMDA increases LC3 II production, which results in the degradation of GluR1, a subunit of AMPAR, in cultured rat hippocampal neurons (Shehata et al., 2012). Treatment with an antagonist of NMDAR, memantine, induces the NMDAR1-mediated autophagic cell death of T-98G cells (Yoon et al., 2017). In cultured hippocampal neurons, the NR2B antagonist Ro25-6981 markedly attenuates NMDA- and global ischaemia-induced activation of the autophagy pathway by disrupting the association of NR2B and Beclin1, resulting in cell death (Borsello et al., 2003; Liu and Zhao, 2013). In contrast, autophagy upregulates the expression of AMPAR subunits, including GluR1, GluR2, and GluR3, in oxygen- and glucose-deprived and reoxygenated injured neurons (Bao et al., 2017). These observations indicate the involvement of Ca^2+^ transporters located in the cell membranes in regulating autophagy. Similarly, VGCC induces Ca^2+^ influx to inhibit autophagy by activating calpains that cleave ATG5, an important factor for elongating autophagosomes, in H4 cells (Williams et al., 2008). As an atypical Ca^2+^ transporter in the cell membrane, APOE4 potentiates the effects of Aβ on the destabilization and permeabilization of lysosomal membranes, which results in impaired autophagy and the degradation of lysosomes in N2a cells (Ji et al., 2006; Nasiri-Ansari et al., 2021). In addition, rapamycin, an autophagy inducer, enhances mitochondrial autophagy and restores mitochondrial function in APOE4-expressing astrocytes (Schmukler et al., 2020). In astrocytes, APOE4 also impairs autophagy, resulting in attenuated clearance of Aβ (Simonovitch et al., 2016; Figure 1).

### ER Stress Induces Autophagy by Modulating the Dyshomeostasis of Ca^2+^

In terms of intracellular Ca^2+^ stores, ER stress induces autophagy in propofol-stimulated C2C12 myoblast cells (Chen et al., 2018). In SK-N-SH cells, ER stress activates autophagy in UPR-stimulated SK-N-SH cells, which indicates its roles in AD (Nijholt et al., 2011). Specifically, polyglutamine induces LC3 conversion *via* ER stress, which initiates the onset of autophagy in C2C5 myoblast cells (Kouroku et al., 2007). Similarly, inducers of ER stress, including tunicamycin, DTT and MG132, concurrently decrease the activity of mTOR and increase the conversion of LC3 I to LC3 II in MEFs (Qin et al., 2010). Lithium induces autophagy by suppressing inositol monophosphatase, leading to the depletion of free inositol and InsP3 in SK-N-SH and COS-7 cells (Sarkar et al., 2005). This observation was also confirmed in lithium-treated IMPA1 knockout mice (Sade et al., 2016). In another study, Ca^2+^ was reported to be located downstream of InsP3R and mediated 2-aminoethoxydiphenyl borate (2-APB)-induced autophagy flux in neonatal rat ventricular myocytes (NRVMs) and HeLa cells (Wong et al., 2013). In addition, by inhibiting InsP3-mediated Ca^2+^ signaling, glucocorticoids induce autophagy in T lymphocytes (Harr et al., 2010). Blockade of InsP3R, the receptor of InsP3, restores autophagy and mitochondrial function in muscle fibers from WT and MDX mice (Valladares et al., 2018). InsP3R knockout upregulates the expression of autophagy markers compared to the WT controls (Cárdenas et al., 2010; Khan and Joseph, 2010). Researchers further emphasized the involvement of Ca^2+^ in autophagy by inducing autophagy through starvation and the activation of the InsP3R-mediated Ca^2+^ signaling pathway, as evidenced by the abolishment of LC3 lipidation and the formation of GFP-LC3 puncta in HeLa cells; these changes were blocked by the Ca^2+^ chelator BAPTA-AM and the InsP3R inhibitor xestospongin B (Cárdenas et al., 2010). In PC12 cells, isoflurane induced autophagy-dependent cell death *via* InsP3R-Ca^2+^-dependent mechanisms (Peng et al., 2011). Moreover, InsP3R-mediated transfer of Ca^2+^ from the ER to mitochondria is required to maintain the proper production of ATP, and Ca^2+^ blockade inhibits AMPK activity, leading to the suppression of autophagy in DT40 cells (Cárdenas et al., 2010; Lim et al., 2021a). Regarding the other Ca^2+^ transporters in ER membranes, RyR mediates the effects of propofol on inducing autophagy in cortical neuronal progenitor cells (Qiao et al., 2017). In primary cultured cortical neurons, RyR1 and RyR3 upregulation induced by insulin deprivation increase Ca^2+^ release from the ER, which increases the production of LC3II, an important autophagy marker (Edinger and Thompson, 2004; Chung et al., 2016). As an antagonist of RyRs, ryanodine stimulates autophagy by decreasing the cytosolic levels of Ca^2+^, leading to neuroprotection in CBE-N2a cells (Liou et al., 2016). By blocking RyR activity, dantrolene and an inhibitory dose of ryanodine reduce the conversion of LC3I to LC3II in HEK293 and C2C12 cells (Vervliet et al., 2017). Similarly, the downregulation of RyR2-mediated Ca^2+^ release decreases ATP production by suppressing mitochondrial metabolism, resulting in an increase in the autophagy-dependent death of rat neonatal cardiomyocytes (Pedrozo et al., 2013; McDaid et al., 2020). By depleting Ca^2+^ from the ER, SOCE exerts a biological effect on Ca^2+^ influx. In PC3 and DU145 cells, autophagic cell death was induced by resveratrol, which downregulated the expression of Stim1 and disrupted its association with TRPC1 and Orai1 (Selvaraj et al., 2016). The overexpression of Stim1 and Orai1 inhibits the effects of starvation- and rapamycin-induced autophagy on A7R5 rat arterial smooth muscle cells (Michiels et al., 2015). Moreover, caerulein promotes the interaction between Stim1 and Orai1, which activates CaN by inducing Ca^2+^ overload, leading to the expression of autophagy-related genes in mice with acute pancreatitis (Zhu et al., 2018). These observations revealed the involvement of ER Ca^2+^ stores in regulating autophagy (Figure 2).

Based on the aforementioned observations, InsP3R was found to connect mitochondria, potentially contributing to apoptosis and autophagy (Decuypere et al., 2011b). In Aβ-treated PC12 cells, moderate activation of autophagy regulates intracellular Ca^2+^ levels and the mitochondrial membrane potential (Xue et al., 2016). Reciprocally, mitochondrial fission-mediated Ca^2+^ signaling induces the expression of Stim1 and subsequent SOCE, which promoted autophagy through Ca^2+^/CAMKK/AMPK signaling cascades (Huang et al., 2017). Regarding Ca^2+^ transporters in mitochondria, VDAC recruits Parkin to defective mitochondria, resulting in the induction of mitochondrial autophagy in HEK293 cells (Sun et al., 2012). In addition, p53 is actively recruited to the outer membrane of mitochondria during nutrient deprivation, resulting in opening of the mPTP, an increase in the conversion of LC3BII to LC3BI, and the formation of LC3-GFP puncta in ventricular myocytes (Eydelnant et al., 2009; Xu H. X. et al., 2020).

### Ca^2+^ Transporters on the Lysosomal Membranes Are Responsible for Regulating the Degradation of Aggregated Proteins

As the lysosome is the organelle responsible for degrading proteins, studies aiming to elucidate the roles of Ca^2+^ transporters located in lysosomes in regulating autophagy would be interesting. For example, Ca^2+^ stimulates lysosomal v-ATPase and mTORC1 pathways, which potentially contribute to the effects of orexin and hypocretin on autophagy in HEK293T cells (Wang et al., 2014). Rapamycin treatment inhibits mTOR activity by decreasing phosphorylation at two serine residues, leading to the induction of autophagy *via* a Ca^2+^-dependent mechanism (Onyenwoke et al., 2015). Furthermore, v-ATPase deficiency in Presenilin 1 (PS1) loss-of-function states causes deficits in lysosomes and autophagy, which contributes to abnormal cellular Ca^2+^ homeostasis (Lee et al., 2015). In addition, accumulating evidence is showing that the functional regulation of TRP channels contributes to Ca^2+^ signaling and subsequent autophagy initiation (Sukumaran et al., 2016). Transient receptor potential cation channel mucolipin subfamily member 1 (TRPML1) is a lysosomal Ca^2+^ channel, which can mediate the release of Ca^2+^ from lysosomes to cytoplasm. TRPML1 mutation increases the formation of autophagosomes, disrupts the fusion of autophagosomes and lysosomes, and induces the accumulation of p62 and insufficient removal of ubiquitinated proteins and/or defective mitochondria in fibroblasts from patients with mucolipidosis type IV (MLIV; Vergarajauregui et al., 2008; Nakamura et al., 2020). Under nutrient starvation conditions, TRPML1 upregulation is critical for increasing lysosomal proteolytic activity in COS-1 cells (Wang W. et al., 2015). Moreover, the overexpression of TRPML3/MCOLN3 induces autophagy in HeLa cells *via* a Ca^2+^-dependent mechanism (Kim et al., 2009). Similarly, both exogenous and endogenous Ca^2+^ modulate autophagy *via* different transporters (Table 5).

**Table 5:** Ca^2+^ disrupts the effects of autophagy on clearing aggregated proteins.

Cat.	Stimulator or mediator	Mechanism	Experimental model	References
Ca^2+^		Mitochondria damage→ROS→TRPML1→Ca^2+^ →autophagy	MCOLN1^−/–^ cells	Zhang et al. (2016)
		Ca^2+^→autophagy	Cardiomyocytes	Shaikh et al. (2016)
CM	NMDAR	Memantine ⊣ NMDAR1 ⊣ autophagic cell death	T-98G cells	Yoon et al. (2017)
		Ro25–6981 ⊣ NMDA ∪ global ischaemia→NR2B ∪ Beclin1→autophagy	Hippocampal neurons	Borsello et al. (2003)
		MiR-93–5p ⊣ PTEN→AKT/mTOR→ NMDA→autophagy	Retinal ganglion cells	Li et al. (2018)
	NMDAR/AMPAR	Low dosage NMDA→LC3 II ⊣ GluR1, a subunit of AMPAR	Rat hippocampal neurons	Shehata et al. (2012)
	AMPAR	Oxygen/glucose-deprived and reoxygenated injured neurons→autophagy→AMPAR, including the subunits of GluR1, GluR2, and GluR3	Primary rat hippocampal neurons	Bao et al. (2017)
	VGCC	VGCC→Ca^2+^ influx→calpains →ATG5 cleavage ⊣ autophagosomes →autophagy	H4 cells	Williams et al. (2008)
	APOE4	APOE4→Aβ→destabilization and permeabilization of lysosomal membranes→degradation of lysosomes ⊣ autophagy	N2a cells	Ji et al. (2006)
		APOE4 ⊣ mitophagy and mitochondrial function	APOE4-expressing astrocytes	Schmukler et al. (2020)
		APOE4 ⊣ autophagy→Aβ clearance	Astrocytes	Simonovitch et al. (2016)
ER		propofol→ER stress→autophagy	C2C12 myoblast cells	Chen et al. (2018)
		UPR→ER stress→autophagy	SK-N-SH cells	Nijholt et al. (2011)
		Polyglutamine→ER stress→LC3 conversion→autophagy	C2C5 myoblast cells	Kouroku et al. (2007)
		Tunicamycin, DTT and MG132→ER stress ⊣ mTOR ∪ →conversion of LC3 I to LC3 II	MEF cells	Qin et al. (2010)
	Ca^2+^	Rapamycin→Ca^2+^ efflux from the ER→autophagy	MCF-7 cells	Høyer-Hansen et al. (2007)
		BAPTA-AM ⊣ Ca^2+^-mobilizing agents→autophagy	MEFs	Grotemeier et al. (2010)
	IP3	Lithium ⊣ inositol monophosphatase→inositol and IP3 ⊣ autophagy	SK-N-SH and COS-7 cells	Sarkar et al. (2005)
		IP3→Beclin1→autophagy	Li-treated IMPA1 KO mice	Sade et al. (2016)
		Glucocorticoids ⊣ IP3→Ca^2+^ efflux from ER ⊣ autophagy	T-lymphocytes	Harr et al. (2010)
	InsP3R	InsP3R ⊣ autophagy	Muscle fibers from WT and MDX mice	Valladares et al. (2018)
		2-aminoethoxydiphenyl borate (2-APB) ⊣ InsP3R→Ca^2+^ release from the ER ⊣ autophagy flux	Neonatal rat ventricular myocytes (NRVMs) and HeLa cells	Wong et al. (2013)
		InsP3R^−/–^→autophagy markers	Chicken DT40B lymphocytes (TKO cells)	Cárdenas et al. (2010) and Khan and Joseph (2010)
		Starvation ⊣ (xestospongin B ⊣ )InsP3R→(BAPTA-AM ⊣ )Ca^2+^ ⊣ LC3 lipidation ∪ GFP-LC3 puncta→autophagy	HeLa cells	Cárdenas et al. (2010)
		Isoflurane ⊣ InsP3R→Ca^2+^ ⊣ autophagic cell death	PC12 cells	Peng et al. (2011)
		InsP3R→Ca^2+^ efflux from the ER→ATP→Ca^2+^ uptake by mitochondria ⊣ AMPK→autophagy	DT40 cells	Cárdenas et al. (2010)
	RyR	ryanodine ⊣ RyRs→autophagy	CBE-N2a cells	Liou et al. (2016)
		Insulin deprivation→RyR1/3→Ca^2+^ efflux from the ER→LC3 II→autophagy	Primary cortical neurons	Edinger and Thompson (2004) and Chung et al. (2016)
		Dantrolene ⊣ RyR→conversion of LC3 I to LC3 II	HEK293 and C2C12 cells	Vervliet et al. (2017)
		RyR^−^ ⊣ Ca^2+^→mitochondrial metabolism→ATP ⊣ autophagic cell death	Rat neonatal cardiomyocytes	Pedrozo et al. (2013)
	SOCE	Resveratrol ⊣ Stim1 ⊣ TRPC1 ∪ Orai1 →autophagic cell death	PC3 and DU145 cells	Selvaraj et al. (2016)
		Stim1^+^ ∪ Orai1^+^ ⊣ starvation ∪ rapamycin →autophagy	A7R5, rat arterial smooth muscle cells	Michiels et al. (2015)
		Caerulein→Stim1 ∪ Orai1→CaN Ca^2+^→autophagy-related genes	Mice with acute pancreatitis	Zhu et al. (2018)
MT	VDAC	VDAC ∪ Parkin→mitochondrial autophagy.	HEK293 cells	Sun et al. (2012)
	mPTP	Nutrient deprivation→p53 ∪ outer membrane of mitochondria→mPTP→conversion from LC3B II to LC3B I ∪ LC3-GFP puncta	Ventricular myocytes	Eydelnant et al. (2009)
LM	v-ATPase	Orexin ∪ hypocretin→v-ATPase→Ca^2+^ influx into lysosomes ∪ mTORC1→autophagy	HEK293T cells	Wang et al. (2014)
		PS1^mut^ ⊣ vATPase→Ca^2+^ influx into lysosomes→autophagy	PS1^mut^ cells	Lee et al. (2015)
	TRPML1	TRPML1^mut^→autophagosomes ∪ ⊣ fusion of autophagosomes and lysosomes→removing p62 and ubiquitinated proteins	Fibroblasts from patients with MLIV	Vergarajauregui et al. (2008)
		Nutrient starvation→TRPML1→lysosomal proteolytic activity	COS-1 cells	Wang W. et al. (2015)
		Rapamycin ⊣ mTOR ⊣ autophagy	HEK293 cells	Onyenwoke et al. (2015)
	TRPML3	TRPML3/MCOLN3^+^→Ca^2+^ →autophagy	HeLa cells	Kim et al. (2009)

## The Herbs Used as Food and Seasonings in Chinese Daily Life Potentially Contribute to AD Treatment by Restoring The Ca^2+^ Concentration Through Effects on Its Transporters

As discussed above, Ca^2+^ overload plays important roles in aggravating AD *via* its transporters. In particular, Ca^2+^ overload perturbs the activities of the brain network, which increases the risk of AD and contributes causally to synaptic and cognitive deficits in hAPP mice. Since Ca^2+^ homeostasis is regulated by different transporters, transporters might be potential therapeutic targets for treating AD by modulating Ca^2+^ homeostasis. However, the outcome is not always consistent with our expectation. For instance, memantine, a noncompetitive NMDA antagonist, is an effective drug approved by the FDA for the treatment of AD. The VGCC inhibitor levetiracetam, an antiepileptic drug, exerts positive effects on patients with AD (Cumbo and Ligori, 2010; Vogl et al., 2012), whereas no beneficial therapeutic effect on AD was observed for the VGCC antagonist nilvadipine (Lawlor et al., 2018).

Although several FDA-approved chemical drugs are currently available for treating AD, the identification of new compounds targeting Ca^2+^ transporters to prevent, halt and reverse the dyshomeostasis of Ca^2+^ is urgently needed. We thereby summarized the drug candidates derived from herbs used as food or seasonings in Chinese daily life used to restore Ca^2+^ homeostasis in animals (Table 6). For example, asiatic acid from *Centella asiatica* reduces intracellular Ca^2+^ levels by inhibiting N- and P/Q-type calcium channels in the rat hippocampus (Lu et al., 2019). In rat cerebrocortical synaptosomes, silymarin derived from *Silybum marianum* similarly reduces intracellular Ca^2+^ concentrations by inhibiting N- and P/Q-type Ca^2+^ channels (Lu et al., 2020a). In addition, the I3C derivative [1(4-chloro-3-nitrobenzenesulfonyl)-1H-indol-3-yl]-methanol (CIM) from broccoli, cauliflower, and brussels sprouts inhibits Ca^2+^ influx by suppressing the activities of P/Q-type Ca^2+^ channels in rats (Lu et al., 2020b). In addition, numerous active compounds, such as uncarialin A, emodin, flavones, aconitine, patchouli alcohol (PA), coutareagenin, neferine, salvianolic acid B (Sal B), danshensu, tetrandrine, osthole, and hydroxy-safflor yellow A, derived from herbs, including *Uncaria rhynchophylla*, rhubarb, *Acanthopanax senticosus* (AS), Aconitum, Cablin, dandelion and *Astragalus*, plantule of *Nelumbo nucifera*, *Salvia miltiorrhiza*, Radix *Salvia miltiorrhiza*, *Stephania tetrandra*, *Cnidium monnieri*, and *Carthamus tinctorius* L., respectively, inhibit Ca^2+^ influx by deactivating Ca^2+^ transporters on the cell membrane, such as L-type Ca^2+^ channels, VDCC, G protein-coupled receptors, TRPCs, and TRPVs in different animal and cell models (Sun G. B. et al., 2014; Vierling et al., 2014; Zhou et al., 2014; Guan et al., 2015; Meng et al., 2016; Yang et al., 2016; Chen R. C. et al., 2017; Li et al., 2018; Yang J. et al., 2020; Yeh et al., 2020; Yu et al., 2020; Yun et al., 2020). Moreover, active compounds, including homoharringtonine, magnolol, polydatin (PD), and *Ginkgo biloba* extracts (EGb), derived from herbs, such as *Cephalotaxus fortunei*, magnolia tree, *Polygonum cuspidatum*, and *Ginkgo biloba*, respectively, modulate Ca^2+^ homeostasis by regulating the activities of transporters located in the ER through mechanism partially dependent on SOCE or mitochondria (Matsubara et al., 2005; Yang et al., 2013; Guo et al., 2014; Hsieh et al., 2018; Li et al., 2019). Although these herbs have not been used in clinical trials, all this evidence suggests that the herbs used as food and seasonings in Chinese daily life potentially contribute to treating AD by targeting Ca^2+^ transporters to restore Ca^2+^ concentrations (Table 6).

**Table 6:** The effects of herbal medicines on regulating Ca^2+^ dyshomeostasis.

Cat.	Herbs	Active compounds	Mechanism	Experimental model	Reference
CM	*Centella asiatica*	Asiatic acid	Asiatic acid ⊣ N- and P/Q-type calcium channels→Ca^2+^ influx	Rat hippocampus	Lu et al. (2019)
	*Silybum marianum*	Silymarin	Silymarin ⊣ N- and P/Q-type Ca^2+^ channels→Ca^2+^ influx	Rat cerebrocortical synaptosomes	Lu et al. (2020a)
	Broccoli, cauliflower and brussels sprouts	I3C derivative [1(4-chloro-3-nitrobenzenesulfonyl)-1H-indol-3-yl]-methanol (CIM)	CIM ⊣ P/Q-type Ca^2+^ channels→Ca^2+^ influx	Rat	Lu et al. (2020b)
	*Uncaria rhynchophylla*	Uncarialin A	Uncarialin A ⊣ L-type calcium channel subunit alpha-1C (Cav1.2)→Ca^2+^ influx	SD rats	Yun et al. (2020)
	Rhubarb	Emodin	Emodin ⊣ L-type Ca^2+^ channels	Isolated beating rabbit atria	Zhou et al. (2014)
	*Acanthopanax senticosus* (AS)	Flavones	Total flavones from AS (TFAS) ⊣ L-type Ca^2+^ channel	SD rats	Guan et al. (2015)
	*Aconitum*	Aconitine	Aconitine→L-type Ca^2+^ channels→intracellular Ca^2+^ levels	Wistar rats	Sun G. B. et al. (2014)
	*Cablin*	Patchouli alcohol (PA)	PA ⊣ VDCC and ROCC→Ca^2+^ influx	Vascular smooth muscle cells (VSMCs)	Li et al. (2018)
	Dandelion and *Astragalus*	Coutareagenin	Coutareagenin ⊣ G protein→Ca^2+^ influx	Rat aortic (A10) cells	Vierling et al. (2014)
	Plantule of *Nelumbo nucifera*	Neferine	Neferine→Gi/o protein ⊣ Ca^2+^ influx	SD rats	Yeh et al. (2020)
	*Salvia miltiorrhiza*	Salvianolic acid B (Sal B)	Sal B ⊣ TRPC3 and TRPC6→intracellular Ca^2+^ levels	Male SD rats	Chen R. C. et al. (2017)
	Radix *Salvia miltiorrhiza*	Danshensu	Danshensu ⊣ p-JNK and NF-κB→TRPC6→Ca^2+^ influx	H9C2 cells	Meng et al. (2016)
	*Stephania tetrandra*	Tetrandrine	Tetrandrine ⊣ RhoA/ROCK pathway→TRPC6→intracellular Ca^2+^ levels	Murine podocytes	Yu et al. (2020)
	*Cnidium monnieri*	Osthole	Osthole ⊣ TRPV1→Ca^2+^ influx	Cultured DRG neurons	Yang et al. (2016)
	*Carthamus tinctorius* L.	Hydroxy-safflor yellow A	HSYA→Endothelial TRPV4→Ca^2+^ influx	Wistar rats	Yang J. et al. (2020)
ER	*Cephalotaxus fortunei*	Homoharringtonine	Homoharringtonine→Histamine H receptor →Ca^2+^ released from the ER→ cytosolic free Ca^2+^ levels	HEK293 cells	Guo et al. (2014)
	Magnolia tree	Magnolol	Magnolol→PKC-sensitive store-operated Ca^2+^→Ca^2+^ influx Magnolol ⊣ endoplasmic reticulum Ca^2+^-ATP pump ⊣ Ca^2+^ release	OC2 cells	Matsubara et al. (2005) and Hsieh et al. (2018)
	*Polygonum cuspidatum*	Polydatin (PD)	PD ⊣ SOCE→intracellular Ca^2+^ levels	Mast cells	Yang et al. (2013)
MT	*Ginkgo biloba*	*Ginkgo biloba* extracts (EGb)	EGb ⊣ mitochondrial Ca^2+^ overload	C57BL/6 mice	Li et al. (2019)

*CM, cell membrane; MT, mitochondria; LM, lysosome; PTM, posttranslational modification; →, stimulate, activate, induce, result in, lead to; ⊣, inhibit, block, suppress, deactivate, degrade; +, overexpress, activate, upregulate, induce; -, knockdown, deplete, ablate, siRNA, deactivate, downregulate, deficiency; −/−, knock out; ∪, interact, facilitate, associate, potentiate, recruit*.

## Conclusions

**Figure 4:**
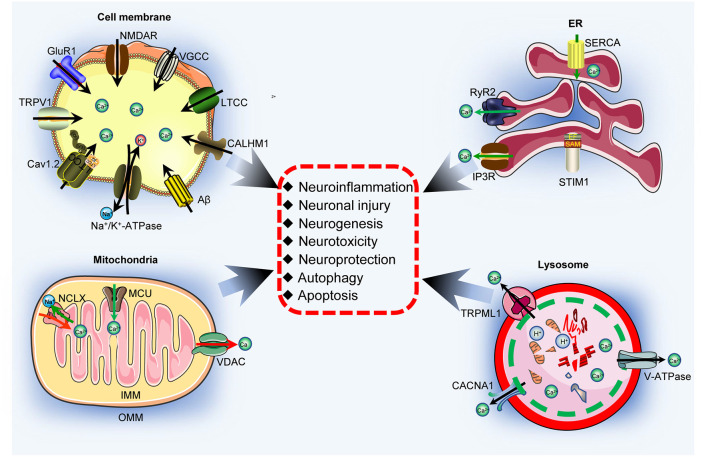
Ca^2+^ transporters are responsible for activating neuronal networks. Ca^2+^ transporters located in the cell membrane, ER, mitochondria, and lysosome are responsible for regulating neuroinflammation, neuronal injury, neurogenesis, neurotoxicity, neuroprotection, autophagy, and apoptosis.

During the development and progression of AD, Ca^2+^ concentrations are increased in the cytosol of neuronal cells *via* transportation from the extracellular space and intracellular stores through transporter-dependent mechanisms. Ca^2+^ accumulation in neuronal cells induces the production and deposition of Aβ and hyperphosphorylated tau in APs and NFTs, leading to impaired learning ability in patients with AD. Moreover, transporters in the cell membrane, endoplasmic reticulum, mitochondria, and lysosomal membranes are critical for mediating the effects of Ca^2+^ on neuroinflammation, neuronal injury, neurogenesis, neurotoxicity, neuroprotection, autophagy, and synaptic plasticity, which contribute to the cognitive decline associated with AD (Figure 4). Based on these theoretical investigations, some bioactive components from Chinese herbal medicines have the potential to treat AD by targeting Ca^2+^ transporters. Moreover, Ca^2+^ transporters are progressively becoming new therapeutic targets for treating AD.

## Author Contributions

P-PG and L-LC contributed to conceptualizing and drafting the manuscript. YY contributed to summarizing the data presented in Table 6. PW contributed to conceptualizing, writing, reviewing, and editing the manuscript. All authors have agreed to publish the manuscript. All authors contributed to the article and approved the submitted version.

## Conflict of Interest

The authors declare that the research was conducted in the absence of any commercial or financial relationships that could be construed as a potential conflict of interest.

## Publisher’s Note

All claims expressed in this article are solely those of the authors and do not necessarily represent those of their affiliated organizations, or those of the publisher, the editors and the reviewers. Any product that may be evaluated in this article, or claim that may be made by its manufacturer, is not guaranteed or endorsed by the publisher.
